# A Fast Synaptic Parameter Estimation Method Based on First- and Second-Order Moments for Short-Term Facilitating Synapses

**DOI:** 10.3390/biomedicines14040771

**Published:** 2026-03-28

**Authors:** Jingyi Zhang, Tianyu Li, Xiaohui Zhang, Liber T. Hua

**Affiliations:** State Key Laboratory of Cognitive Neuroscience and Learning, IDG-McGovern Institute for Brain Research, Faculty of Psychology, Beijing Normal University, Beijing 100875, China; joy_zhang@mail.bnu.edu.cn (J.Z.); 202031061042@mail.bnu.edu.cn (T.L.); xhzhang@bnu.edu.cn (X.Z.)

**Keywords:** synaptic transmission, release probability, mean–variance method, nonlinear calcium dynamics

## Abstract

**Background**: Short-term facilitation (STF) is a key form of synaptic plasticity driven by activity-dependent increases in presynaptic release probability. However, estimating core synaptic parameters—quantal size (q), vesicle pool size (N), and release probability ($p_{i}$)—remains challenging due to nonlinear dynamics and unobservable presynaptic states, limiting the applicability of conventional methods. **Methods**: We developed a fast analytical framework based on first- and second-order statistical moments of evoked EPSCs, including mean, variance, and cross-stimulus covariance. By constructing composite moment relationships, latent variables were algebraically eliminated, yielding closed-form estimators of synaptic parameters. To improve robustness under strong facilitation, a Tsodyks–Markram (T–M) model-based calibration step was introduced to refine N and $p_{i}$ using the estimated q as a constraint. **Results**: Applied to hippocampal CA3–CA1 synapses, the method produced accurate and stable estimates of q across varying noise and sampling conditions. Incorporation of cross-stimulus covariance enabled effective characterization of structured variability that is neglected in classical approaches. While direct estimates of N and $p_{i}$ showed dispersion, T–M calibration significantly improved stability and physiological consistency. Compared with mean–variance analysis, the proposed method achieved superior performance under facilitating conditions. **Conclusions**: This hybrid framework enables rapid and reliable estimation of synaptic parameters in STF synapses by exploiting second-order statistical structure. It provides a practical tool for investigating presynaptic mechanisms and may facilitate quantitative studies of synaptic dysfunction in neurological and psychiatric disorders.

## 1. Introduction

Synaptic transmission is not a static relay of information but a dynamic process that continuously adapts to recent activity. Short-term synaptic plasticity (STP), operating on timescales from milliseconds to minutes, plays a critical role in shaping neural information processing. By modulating synaptic efficacy in an activity-dependent manner, STP influences signal propagation, temporal filtering, and network dynamics [1,2,3,4].

STP is commonly described as the interplay between short-term depression and short-term facilitation. Facilitation, in particular, is prominent at synapses with low initial release probability and enables transient amplification of synaptic responses during repetitive stimulation. This mechanism is thought to support burst detection, frequency-dependent signal transmission, and selective routing of information in cortical and subcortical circuits [1,2,5]. However, facilitating synapses are not governed by a single process. Instead, facilitation driven by residual presynaptic ${Ca}^{2+}$ accumulation coexists with vesicle depletion, replenishment, and priming, leading to complex, stimulus-dependent dynamics across response trains [1,5,6].

Importantly, alterations in short-term synaptic plasticity have been increasingly implicated in neurological and psychiatric disorders. Changes in presynaptic release probability, vesicle pool dynamics, and ${Ca}^{2+}$-dependent facilitation have been reported in conditions such as schizophrenia, major depressive disorder, epilepsy, and Parkinson’s disease, where they are thought to contribute to disrupted synaptic signaling and circuit dysfunction [7,8,9,10]. Because STP directly shapes how neurons process temporal patterns of activity, even subtle changes in presynaptic parameters can lead to significant alterations in network behavior. This makes reliable quantification of synaptic release parameters not only a fundamental problem in synaptic physiology, but also a potentially important step toward understanding disease-related synaptic dysfunction.

From a quantitative perspective, synaptic transmission is often described in terms of a small set of parameters: the quantal size q, representing the postsynaptic response to a single vesicle; the effective number of release sites or vesicle pool size N; and the stimulus-dependent release probability p. These parameters provide a compact statistical description linking presynaptic mechanisms to postsynaptic responses. However, in facilitating synapses, these quantities are not static. Instead, they evolve dynamically across stimulus trains and may reflect composite effects of vesicle occupancy, priming state, and fusion probability rather than direct anatomical measures [5,11,12,13].

In many synapses, neurotransmitter release is commonly described as a quantal process, in which each presynaptic action potential triggers the probabilistic release of discrete vesicles [14,15]. Within this framework, synaptic responses can be modeled as the aggregate outcome of a finite number of release sites, each releasing a vesicle with a certain probability [16,17]. This leads naturally to a binomial description [10,18], where the mean and variance of postsynaptic responses are determined by the quantal size q, the effective number of release sites N, and the release probability p  [16]. Such a formulation provides a convenient statistical bridge between experimentally observed postsynaptic currents and the underlying presynaptic mechanisms [9,18].

However, not all variables governing synaptic transmission are directly observable in electrophysiological recordings. In particular, quantities such as the instantaneous release probability, vesicle occupancy, or internal calcium-dependent states evolve dynamically during stimulus trains but cannot be measured directly from postsynaptic responses. These hidden or latent variables introduce ambiguity into parameter estimation, because multiple combinations of underlying states can give rise to similar observable statistics. As a result, reliable inference requires either explicit modeling of these hidden processes or the use of additional statistical constraints that allow them to be eliminated or indirectly inferred.

Rather than explicitly fitting all hidden presynaptic states, we take advantage of the statistical structure of synaptic responses. By jointly analyzing first- and second-order moments, including cross-stimulus covariance, our approach provides sufficient constraints to eliminate latent variables analytically and directly estimate key synaptic parameters.

Classical approaches for estimating synaptic parameters, such as mean–variance analysis and related fluctuation-based methods, have proven effective under relatively stationary conditions [11,12,13]. However, their application to facilitating synapses remains challenging. First, release probability varies strongly across stimuli. Second, facilitation introduces nonlinear, ${Ca}^{2+}$-dependent dynamics. Third, trial-to-trial fluctuations across different stimuli are often structured, such that correlations between responses contain information that is not captured by the variance of individual responses alone. As a result, methods that rely solely on first-order statistics or treat responses independently may fail to capture key aspects of synaptic dynamics.

## 2. Materials and Methods

**Figure 1:**
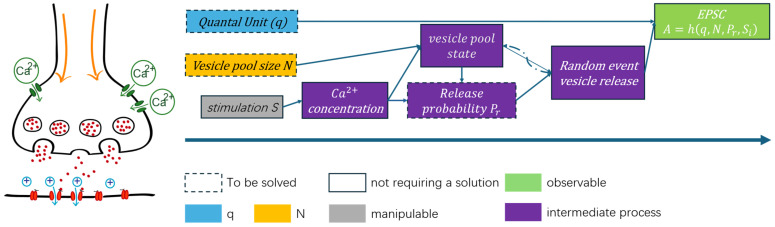
Simplified principle model of synaptic transmission process in synaptic plasticity. On the left, green circles represent voltage-gated calcium channels (VGCCs) in the presynaptic membrane. Upon arrival of an action potential (indicated by an orange arrow), these channels open, allowing an influx of extracellular ${Ca}^{2+}$ ions (depicted as red dots). The resulting rise in intracellular ${Ca}^{2+}$ concentration serves as the primary trigger for vesicular neurotransmitter release. Below, synaptic vesicles (in red) are shown within the presynaptic vesicle pool, along with their mobilization toward the active zone and subsequent fusion with the presynaptic membrane. These factors collectively govern the stochastic process of vesicle release, ultimately determining the amplitude A of the EPSC, modeled as $A=h\left(q,N,P_{r},S_{i}\right)$, where $S_{i}$ may denote a time-varying stimulus or internal state variable. Color-coded boxes highlight distinct components: blue for the quantal unit (q), yellow for vesicle pool size (N), gray for stimulation (S), purple for ${Ca}^{2+}$ dynamics and release probability ($P_{r}$), and green for the resulting EPSC output. Together, this framework captures the quantal nature of synaptic transmission and its sensitivity to activity-dependent modulation, providing a foundation for modeling both short-term (e.g., facilitation, depression) and long-term forms of synaptic plasticity. The synapse illustration was created by Peter Kraemer [19].

The synaptic release framework underlying the short-term synaptic plasticity parameters is schematically illustrated in Figure 1. Neurotransmitter release is modeled as a quantal process, where each vesicle contributes a fixed quantal unit q. The amplitude of the excitatory postsynaptic current (EPSC) is determined by the interaction between vesicle pool size N, release probability $p_{i}$, and stimulation S. Presynaptic stimulation elevates intracellular ${Ca}^{2+}$ concentration, which modulates the vesicle pool state and dynamically regulates $P_{r}$. Vesicle fusion is treated as a stochastic event governed by these state variables. Accordingly, synaptic efficacy is formalized as $A=h\left(q,N,P_{r},S_{i}\right)$, linking measurable postsynaptic responses to presynaptic quantal parameters and calcium-dependent release dynamics.

While Figure 1 captures the observable structure of synaptic responses under repetitive stimulation, it is important to note that several underlying presynaptic processes are not directly accessible from electrophysiological recordings. In particular, variables such as instantaneous release probability, vesicle occupancy within the readily releasable pool, and calcium-dependent internal states evolve dynamically across stimuli but remain unobserved in postsynaptic measurements.

These hidden or latent variables play a critical role in shaping synaptic responses, yet they introduce substantial ambiguity for parameter estimation, as different configurations of these internal states can give rise to similar observable EPSC statistics. Explicitly modeling all such latent processes would require detailed assumptions about their dynamics and interactions, often leading to increased model complexity and reduced robustness in practical data fitting.

In this study, rather than attempting to explicitly reconstruct these latent variables, we focus on exploiting the statistical structure of observable responses. By leveraging first- and second-order moments, including cross-stimulus covariance, our approach provides sufficient constraints to effectively eliminate the influence of latent variables and directly estimate key synaptic parameters.

### 2.1. Experimental Data Acquisition and Preprocessing

#### 2.1.1. Hippocampal Slice Preparation

This study utilized three male C57/BL wild-type mice at postnatal days 35–45. The mice were anesthetized by i.p. injection of sodium pentobarbital (50 mg/kg of b.w.) and then perfused with the cold, oxygenated sucrose-substituted artificial cerebrospinal fluid (sucrose aCSF, 15 mL), composed of (in mM) 125 sucrose, 1.25 NaH_2_PO_4_, 2 CaCl_2_, 3 KCl, 2 MgSO_4_, 1.3 sodium ascorbate and 0.6 sodium pyruvate (pH 7.30, 305 mOsm). After decapitation, the brain was quickly dissected and transferred into the oxygenated sucrose-substituted aCSF (at 0 °C). Coronal sections of the hippocampus in 300 μm thickness were made with a vibratome (VT1200, Leica Nussloch, Germany) in the sucrose aCSF at 0 °C. The hippocampal slices were then transferred into an incubation chamber filled with the normal aCSF (in mM, 125 NaCl, 1.25 NaH_2_PO_4_, 2 CaCl_2_, 3 KCl, 2 MgSO_4_, 1.3 sodium ascorbate and 0.6 sodium pyruvate; pH 7.30, 305 mOsm), oxygenated with 95% O_2_/5% CO_2_ for 30 min at 34 °C, followed by incubation at room temperature (25 ± 2 °C) for more than 30 min before recording.

#### 2.1.2. Slice Electrophysiology Recording

Hippocampal slices were transferred to a recording chamber perfused with the oxygenated aCSF containing a selective GABAA receptor antagonist picrotoxin (PTX, 50 μM) and an NMDA-type glutamate receptor inhibitor D-AP5 (50 μM) at room temperature. Whole-cell voltage clamp recordings of excitatory pyramidal cells (PCs) in the stratum pyramidal (s.p.) layer of hippocampal CA1 region were made to record evoked excitatory postsynaptic current (EPSCs), with a MultiClamp 700B amplifier (Molecular Devices, Sunnyvale, CA, USA), under a Nikon FN1 (Nikon, Tokyo, Japan) upright microscopy. Recording micropipettes, with a tip size 2–3 μm, were made from borosilicate glass capillaries (B-120-69-15, Sutter, Novato, CA, USA) with a Sutter puller (P-1000) and filled with an internal solution composed of (in mM) 130 K-D-gluconate, 20 KCl, 10 Hepes, 10 Disodium phosphocreatine, 4 Mg2ATP, 0.3 Na2GTP, 0.2 EGTA (pH 7.32, 299 mOsm). Micropipette resistances were in the range of 3–6 MΩ. Electric signals were digitalized by a Digidata 1440A converter (Molecular Devices, Sunnyvale, CA, USA), and acquired at 20 kHz by PClamp 10.3 (Molecular Devices, Sunnyvale, CA, USA) for further analysis. All chemicals were from Sigma-Aldrich (St. Louis, MO, USA).

#### 2.1.3. Data Preprocessing

To evoke the synaptic transmission of CA3-CA1 Schaffer collateral (SC) synapses, a concentric tungsten electrode (MCE-100; Rhodes Medical Instruments, Woodland Hills, CA, USA) was placed in the CA1 stratum radiatum (s.r.) layer to excite CA3-SC axonal fibers (at the depth of 50–80 μm in the tissue) in a position 100–200 μm horizontally away from the recorded PCs. It should be noted that a cut of the slice between CA3 and CA1 were made to eliminate most CA3 inputs and repetitive firing caused by reduced GABAergic inhibition. Ten electric pulses with 1 ms duration were delivered at 20, 50, or 75 Hz, via an Isofex-100 isolator (A.M.P. Instruments, Jerusalem, Israel). The stimulation intensities ranged from 0.01 to 2 mA, with the first evoked EPSCs in a range of 10–50 pA on average for each examined slice. In the presence of PTX (50 μM) and the NMDA glutamate receptor antagonist AP5 (50 μM), evoked EPSCs recorded at a clamped membrane potential of −70 mV were mainly mediated by AMPA-type glutamate receptors, and they were averaged by twenty sweeps at each stimulation frequency.

The EPSC peak analysis was performed using custom MATLAB scripts. The recorded signals in .abf format were imported, and segments following each stimulus were extracted to capture individual EPSC responses. A median filter (medfilt1 function) was applied to smooth the electrophysiological data. The amplitude of each EPSC was calculated as the difference between the baseline immediately after the stimulus artifact and the first subsequent peak. The baseline was defined as the signal current prior to the onset of the stimulus train. For each stimulus, the EPSC peak was identified as the maximum current value within the interval between two consecutive stimuli. This procedure ensures consistent extraction of peak amplitudes across the stimulus sequence.

Cells were excluded based on the following quality control criteria: (1) series resistance exceeding 30 MΩ or resting membrane potential above −60 mV; (2) a loss of voltage clamp integrity during recording, and (3) fewer than 20 valid sweeps per recording condition.

### 2.2. Assumptions and Simplifications

To enable rapid and robust estimation of synaptic parameters from limited experimental data, several assumptions and simplifications are introduced. These assumptions are guided by established quantal models of synaptic transmission.

#### 2.2.1. Independence of Stochastic Components

We assume that the three stochastic components contributing to EPSC variability—vesicle release number N, quantal size q, and measurement noise $\eta_{i}$—are statistically independent. In particular, measurement noise is assumed to be independent across stimuli and sweeps, with zero mean and constant variance. This assumption allows noise-related terms to vanish in first- and second-order moment calculations and simplifies the analytical expressions for covariance and variance.

#### 2.2.2. Binomial Release Model with Effective Pool Size

Synaptic vesicle release is modeled as a binomial process with parameters N and $p_{i}$. For facilitating synapses, the total vesicle pool is typically large, and depletion does not significantly affect the first few responses in a stimulus train. Therefore, the parameter N is interpreted as an $N_{effect}$ number of release sites participating during the initial phase of stimulation rather than the full anatomical vesicle pool.

Because inactive or reserve vesicles do not contribute to release during this early phase, they cannot be inferred from electrophysiological data. As a result, estimates of N obtained in this framework are expected to underestimate the true vesicle pool size. In practice, when multiple estimates of N are obtained from different stimulus index combinations, the maximal stable value is taken as the most biologically plausible estimate.

#### 2.2.3. Stimulus-Dependent Release Probability as a Latent Variable

The release probability $p_{i}$ is allowed to vary with stimulus index i, reflecting short-term facilitation driven primarily by presynaptic calcium accumulation. Biophysically, $p_{i}$ represents the product of (i) the probability that a release site is occupied by a vesicle and (ii) the probability that a vesicle is released given occupancy.

Because these two factors cannot be reliably separated from EPSC amplitude data alone—especially under dynamic calcium conditions—we treat their product as a single effective release probability. The detailed calcium dynamics underlying facilitation are not explicitly modeled; instead, $p_{i}$ is treated as a latent, stimulus-dependent variable that will later be eliminated using observable moment relationships.

#### 2.2.4. Quantal Size Variability Across Sweeps

We allow the quantal size q to vary across experimental sweeps to account for slow fluctuations in postsynaptic responsiveness, receptor state, or recording conditions. Within a single sweep, however, $q_{sweep}$ is assumed to be constant, such that all vesicles released during the same stimulus contribute equally to the EPSC amplitude.

Depending on data quality and experimental conditions, two regimes are considered in later analysis: (i) negligible quantal variability across sweeps, enabling a rapid approximation scheme, and (ii) non-negligible quantal variability, which requires explicit incorporation of second-order moment information.

### 2.3. Synaptic Transmission Model

To quantitatively describe synaptic transmission during short-term facilitation, we adopt a probabilistic generative model for excitatory postsynaptic currents (EPSCs) evoked by stimulus trains. The model integrates classical quantal analysis with stimulus-dependent vesicle release statistics, providing a compact yet flexible framework for subsequent moment-based parameter estimation.

A detailed derivation is given in the Supplementary Materials (Section S1), to which the reader is referred for completeness.

#### 2.3.1. EPSC Generative Model

We assume that the amplitude of the EPSC evoked by the i-th stimulus in a stimulus train is a random variable, denoted as $A_{i}$ (unit: pA). For a given synapse, the EPSC amplitude in each sweep is modeled as the linear superposition of released synaptic vesicles, combined with measurement noise:

(1) $$A_{i}=q_{sweep}\cdot k_{i}+\eta_{i}$$

where $q_{sweep}$ represents the quantal size, defined as the postsynaptic current contribution of a single vesicle, $k_{i}$ is the number of vesicles released in response to the i-th stimulus, and $\eta_{i}$ denotes additive measurement noise, following a normal distribution with standard deviation $\sigma_{\eta }$.

(2) $$\eta_{i}\sim Normal(0,\sigma_{\eta }^{2})$$

The quantal size $q_{sweep}$ is allowed to vary across experimental sweeps to account for biological variability in postsynaptic responsiveness, while its fluctuation within a single sweep is assumed to be negligible. Measurement noise $\eta_{i}$ is modeled as an independent Gaussian random variable with zero mean, reflecting baseline noise recorded during resting condition.

It should be noted that the postsynaptic response $A_{i}$ can equivalently be expressed in terms of excitatory postsynaptic potential (EPSP) amplitude, (unit: mV), particularly when voltage clamp conditions are not imposed or when analyzing subthreshold membrane potential dynamics. In such cases, the same stochastic framework applies, with $A_{i}$ reflecting the summed contribution of quantal EPSPs and background fluctuations.

When the postsynaptic response is measured as an EPSP instead of an EPSC, the quantal size q must also be expressed in voltage units—specifically, millivolts (mV)—to maintain consistency. In this case, the total EPSP amplitude $A_{i}$ is modeled as the sum of released quanta, each contributing q (in mV), plus noise.

#### 2.3.2. Vesicle Release Statistics

Vesicle release is modeled using a classical binomial framework. Specifically, the number of released vesicles $k_{i}$ follows a binomial distribution:

(3) $$k_{i}\sim Binomial(N,p_{i})$$

where N denotes the effective number of release sites participating in synaptic transmission during the initial phase of the stimulus train, and $p_{i}$ is the stimulus-dependent release probability associated with the i-th pulse.

### 2.4. Moment-Based Statistical Characterization of EPSC

To enable fast and robust estimation of synaptic parameters without explicitly solving the full stochastic release dynamics, we characterize the EPSC responses evoked by stimulus trains using first- and second-order statistical moments computed across repeated sweeps from the same synapse. All moment estimates are obtained at the single-cell level and are therefore intrinsic to individual synaptic terminals.

Let $A_{i}$ denote the EPSC amplitude evoked by the i-th stimulus in a train. For each stimulus index i, EPSC responses are recorded across repeated sweeps, forming a random variable whose statistical properties reflect both presynaptic release variability and postsynaptic quantal fluctuations.

From a statistical perspective, EPSC amplitudes $A_{i}$ are treated as random variables across repeated sweeps. For each stimulus index i, the collection of EPSC amplitudes forms a distribution that reflects both presynaptic release variability and postsynaptic quantal fluctuations.

Within this framework, different statistical moments capture distinct aspects of synaptic variability. The mean represents the average synaptic response at each stimulus index, while the variance quantifies trial-to-trial variability arising from release statistics, quantal fluctuations, and measurement noise. Importantly, the covariance between responses at different stimulus indices captures shared fluctuations across sweeps, indicating structured variability beyond independent noise.

This structured statistical dependence provides the basis for the moment-based analysis developed here, allowing for informative constraints on synaptic parameters without explicitly modeling latent variables.

#### 2.4.1. First-Order Moment (Mean EPSC)

The first-order moment of the EPSC distribution is defined as the mean response across sweeps for a given stimulus index:

(4) $$\mu_{i}=E\left(A_{i}\right)$$

Under the generative model introduced above, the EPSC amplitude is expressed as the product of the number of released vesicles and the quantal size, with an additive measurement noise term. Because the measurement noise is assumed to be zero-mean and independent across sweeps, it does not contribute to the expectation value. Consequently, the mean EPSC depends only on the expected vesicle release count and the mean quantal size.

The mean response thus provides a direct observable that captures the effective synaptic strength at each stimulus position within the train. Importantly, for facilitating synapses, the temporal evolution of $\mu_{i}$ reflects stimulus-dependent changes in release probability, while remaining agnostic to the detailed biophysical mechanisms underlying facilitation.

#### 2.4.2. Second-Order Moment: Covariance

**Figure 2:**
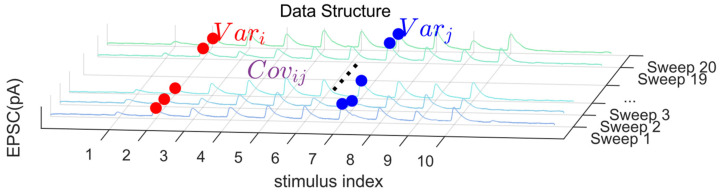
An example diagram of data structure. The data in this diagram comes from 20 sweeps, with 10 electrical stimulations per sweep. In order to simplify the drawing, we have only drawn some of the data to facilitate readers’ understanding of the principle. The peak amplitude after each EPSC stimulation is extracted from the raw electrophysiological trace. Specifically, the baseline is defined as the signal current prior to the onset of the stimulus train. For each stimulus index i, the EPSC peak is defined as the maximum current value within the time window between the i-th stimulus and the subsequent (i+1)-th stimulus. The peak amplitude is then calculated relative to this baseline. The EPSC peak amplitudes from repeated sweeps are grouped by stimulus index. The amplitudes of the i-th stimulus across sweeps are used to compute variance, while the relationship between the i-th and j-th stimuli is used to compute covariance.

To clarify the definition of stimulus indices used in the covariance calculation, we refer to the schematic illustration in Figure 2. In repetitive stimulation experiments, synaptic responses are typically recorded as sequences of evoked EPSCs across multiple trials. Within each trial, stimuli are delivered in a fixed temporal order, and each evoked response can be indexed according to its position in the stimulus train.

Specifically, we denote the response to the k-th stimulus within a trial as $A_{k}$, where k = 1, 2, ..., K, and K is the total number of stimuli in a train. Across repeated trials, responses corresponding to the same stimulus index k are grouped together to compute their statistical properties. The covariance considered in this study is therefore defined between responses at different stimulus positions (i and j) across trials, i.e., between $A_{i}$ and $A_{j}$ measured at the same relative positions within each stimulus train.

As illustrated in Figure 2, this organization allows us to construct cross-stimulus covariance matrices that capture structured trial-to-trial variability across the stimulus sequence. Such covariance contains additional information beyond single-stimulus variance and plays a key role in constraining synaptic parameters in our framework.

To extract additional information beyond mean dynamics, we next consider the second-order cross-moment between EPSC responses evoked by two distinct stimuli i and j:

(5) $$Cov\left(A_{i},A_{j}\right)=E[\left(A_{i}-\mu_{i}\right)\cdot \left(A_{j}-\mu_{j}\right)]$$

Because measurement noise is assumed to be independent across stimuli and sweeps, it does not contribute to the covariance term. The covariance therefore arises solely from shared fluctuations in presynaptic and postsynaptic variables across repeated trials.

In particular, the covariance captures sweep-to-sweep co-variability induced by trial-dependent quantal size fluctuations and shared release statistics. This property makes covariance a powerful observable for eliminating latent variables that are otherwise difficult to estimate directly, such as stimulus-dependent release probability. By combining covariance with first-order moments, stimulus-specific parameters can be algebraically eliminated in later estimation steps.

Notably, non-zero covariance between responses at different stimulus indices provides direct empirical evidence that EPSC fluctuations are structured across sweeps rather than dominated by uncorrelated noise.

#### 2.4.3. Second-Order Moment: Variance

When the two indices coincide (i=j), the second-order moment reduces to the variance of the EPSC distribution:

(6) $$Var(A_{i})=E[Var(A_{i}|q_{sweep})]+Var[E(A_{i}|q_{sweep})]$$

The variance reflects contributions from multiple sources of variability, including binomial vesicle release statistics, quantal size fluctuations across sweeps, and measurement noise. To properly account for these components, the variance is interpreted through the law of total variance, which separates within-sweep variability from between-sweep variability.

Unlike the covariance, the variance explicitly contains contributions from stimulus-dependent release probability, making it sensitive to both facilitation dynamics and quantal variability. While this dependence complicates direct parameter inference, the variance becomes highly informative when combined with covariance-based quantities that allow for selective elimination of unobservable terms.

Together, the mean, covariance, and variance constitute a complete set of low-order statistical descriptors that can be robustly estimated from experimental data and jointly constrain the effective synaptic parameters of interest.

### 2.5. Parameter Elimination Strategy Based on Observable Moments

The expressions derived above still contain several latent variables that are either stimulus-dependent or biologically inaccessible, such as the instantaneous release probability $p_{i}$ and the quadratic term $N^{2}\cdot p_{i}^{2}$. Direct estimation of these quantities is numerically unstable and experimentally impractical. Therefore, we next introduce a sequence of algebraic simplifications that systematically eliminate these irretrievable relationships while preserving all information accessible from observable moments.

#### 2.5.1. Elimination of $N^{2}\cdot p_{i}^{2}$

Inspection of the variance expression reveals that the dominant high-order latent contribution arises from the term proportional to $N^{2}\cdot p_{i}^{2}$, which originates from sweep-to-sweep variability of the quantal amplitude. Although this term cannot be directly computed, it also appears in the covariance between responses at different stimulus indices. This redundancy allows us to remove it by forming an appropriate linear combination of second-order moments.

Specifically, we define a fully observable composite quantity

(7) $$F_{i,j}=Var(A_{i})-\frac{\mu_{i}}{\mu_{j}}Cov\left(A_{i},A_{j}\right)-\sigma_{\eta }^{2},$$

which depends only on empirically measurable means, variances, covariances, and baseline noise. By construction, this combination exactly cancels the $N^{2}\cdot p_{i}^{2}$ contribution, leaving a simplified expression that depends solely on first-order binomial terms.

After simplification, $F_{i,j}$ reduces to

(8) $$F_{i,j}=E\left(q_{sweep}^{2}\right)\cdot N\cdot p_{i}\cdot \left(1-p_{i}\right),$$

where $E\left(q_{sweep}^{2}\right)$ represents the second moment of the quantal amplitude across sweeps. Importantly, this result no longer contains any quadratic dependence on N or $p_{i}$, making it substantially more tractable for parameter inference.

#### 2.5.2. Elimination of the Stimulus-Dependent Release Probability $p_{i}$

Our primary objective is to estimate the quantal size q without relying on stimulus-specific release probabilities. To this end, we substitute the mean–current relationship $\mu_{i}=q\cdot N\cdot p_{i}$ into the expression for $F_{i,j}$, thereby rewriting it entirely in terms of observable quantities and global parameters.

This transformation yields an explicit linear relation between $F_{i,j}$, $\mu_{i}$, q, and N. By forming two such equations using distinct stimulus indices, the unknown pool size N can be algebraically eliminated. As a result, the quantal amplitude q admits a closed-form solution expressed purely in terms of measured means and the composite observables $F_{i,j}$.

Crucially, this step removes all dependence on the unobservable release probability sequence $p_{i}$, allowing q to be inferred directly from second-order statistics.

### 2.6. Treatment of Quantal Variability

Our primary goal is to obtain a closed-form estimator for the quantal size q without explicitly tracking the stimulus-indexed release probability $p_{i}$. Leveraging the observable statistic $F_{i,j}$ together with the empirical means $\mu_{i}$, we can obtain a closed-form solution for q using any valid index tuple (i, j, k,l) (with distinctness constraints as specified later). The resulting estimator takes the form

(9) $$q=\frac{\mu_{k}\mu_{i}E\left(q_{sweep}^{2}\right)(\mu_{k}-\mu_{i})}{\mu_{k}^{2}F_{i,j}-\mu_{i}^{2}F_{k,l}}.$$

#### Handling the Within-Sweep Quantal Variability Term $\sigma_{q}^{2}$

When the quantal unit is sufficiently stable across sweeps (i.e., $\sigma_{q}^{2}\to 0$), the second moment can be approximated as $E\left(q_{sweep}^{2}\right)\approx q^{2}$. Under this assumption, the quantal size admits an explicit closed-form approximation:

(10) $$q\approx \frac{\mu_{k}^{2}F_{i,j}-\mu_{i}^{2}F_{k,l}}{\mu_{k}\mu_{i}(\mu_{k}-\mu_{i})}$$

Given this q, the remaining parameters follow immediately:

(11) $$N\approx \frac{\mu_{i}^{2}}{\mu_{i}q-F_{i,j}}$$

(12) $$\hat{p_{i}}=\frac{\mu_{i}}{qN}$$

This branch is intended for fast screening or for datasets where sweep-to-sweep quantal variability is empirically minimal.

When sweep-to-sweep quantal variability is non-negligible, we estimate the ratio

(13) $$R_{i,j}=\frac{\sigma_{q}^{2}}{q^{2}}=\frac{Cov\left(A_{i},A_{j}\right)}{\mu_{i}\cdot \mu_{j}}$$

and obtain a robust estimate $\hat{R}$ by averaging across all valid index pairs (i,j) in the dataset:

(14) $$\hat{R}=E(\frac{Cov\left(A_{i},A_{j}\right)}{\mu_{i}\cdot \mu_{j}})$$

In the previous derivation, we obtained closed-form estimators under the simplifying assumption that the quantal size q is constant across sweeps. However, in real experimental recordings, the quantal amplitude typically exhibits trial-to-trial (sweep-level) variability due to biological and measurement factors.

This variability introduces additional contributions to second-order statistics (variance and covariance), which, if ignored, can bias parameter estimation. Therefore, we next extend the estimator to explicitly account for quantal variability in a fully observable manner.

To incorporate this effect, we express the second moment of the quantal amplitude as follows:

(15) $$E\left(q_{sweep}^{2}\right)={\hat{q}}^{2}\cdot (1+\hat{R})$$

where $\hat{R}$ represents the normalized quantal variability. Importantly, $\hat{R}$ can be estimated directly from experimentally measured covariance and mean values (see Equations (13) and (14)), making this correction fully data-driven.

Substituting this expression into the previous estimator yields a corrected closed-form solution for the quantal size:

(16) $$\hat{q}=\frac{\mu_{k}^{2}F_{i,j}-\mu_{i}^{2}F_{k,l}}{\mu_{k}\mu_{i}(1+\hat{R})(\mu_{k}-\mu_{i})}$$

Compared to the simplified estimator (Equation (10)), this expression explicitly compensates for quantal variability through the factor $\left(1+\hat{R}\right)$.

As a result, $\hat{q}$ remains robust even when sweep-to-sweep fluctuations in quantal amplitude are non-negligible.

Once the corrected quantal size $\hat{q}$ is obtained, the effective vesicle pool size can be estimated as follows:

(17) $$\hat{N}=\frac{\mu_{i}^{2}}{\mu_{i}\hat{q}-\frac{F_{i,j}}{\left(1+\hat{R}\right)}}$$

(18) $$\hat{p_{i}}=\frac{\mu_{i}}{\hat{q}\hat{N}}$$

This expression shows that the estimation of N directly depends on the corrected second-order statistics.

In particular, the same variability correction factor $\left(1+\hat{R}\right)$ appears here, ensuring consistency between the estimation of q and N.

Taken together, Equations (15)–(17) form a sequential estimation procedure:

Equation (15) estimates the contribution of quantal variability using observable covariance.

Equation (16) uses this correction to obtain an unbiased estimate of the quantal size q.

Equation (17) propagates the corrected statistics to estimate the vesicle pool size N.

This sequence ensures that variability originating from quantal fluctuations is not misattributed to synaptic release parameters, thereby improving both accuracy and robustness. Conceptually, this step can be understood as separating intrinsic synaptic variability from measurement-induced fluctuations, similar to variance decomposition in statistical modeling.

### 2.7. Parameter Optimization and Post-Processing

Using the closed-form expressions, we directly estimate synaptic parameters from empirical first- and second-order moments. For any admissible index tuple (i,j,k,l) satisfying i≠j, i≠k and k≠l, candidate estimates of the quantal size $\hat{q}$, effective pool size N, and release probability $p_{i}$ are computed.

To improve robustness against experimental noise, multiple $\hat{q}$ estimates are obtained by enumerating all valid index tuples. Negative or non-physical values are treated as noise-induced outliers and removed. The remaining estimates are then averaged to yield the final quantal size estimate $E(\hat{q})$.

For the estimation of N, covariance-based fluctuations and saturation effects may introduce downward bias. To mitigate this, extreme $\hat{N}$ values are excluded using a 3σ criterion based on the empirical distribution of candidates. Among the remaining values, we select the maximum as the final estimate $\hat{N}$, reflecting the effective size of the readily releasable pool. Once $E(\hat{q})$ and $\hat{N}$ are fixed, the release probability $\hat{p_{i}}$ is recomputed accordingly.

(19) $$\left\{\begin{array}{c} E\left(\hat{q}\right)=\frac{1}{C_{T}^{2}\cdot C_{T-1}^{2}}\sum_{i\neq j}^{C_{T}^{2}}\sum_{k\neq i\;\&\;k\neq l}^{C_{T-1}^{2}}{\hat{q}}_{i,j,k,l}\\ \hat{N}=\underset{{\hat{N}}_{i,j}\in \mu \pm 3\sigma }{\max}{\hat{N}}_{i,j}\\ \hat{p_{i}}=\frac{\mu_{i}}{\hat{q}\hat{N}} \end{array}\right.$$

### 2.8. Tsodyks–Markram Model-Based Calibration of N and $p_{i}$

Although the proposed moment-based elimination framework provides robust closed-form estimates of the quantal size q, the estimation of the effective vesicle pool size N and stimulus-dependent release probability $p_{i}$ remains sensitive to the binomial assumption. In particular, when synaptic facilitation is strong, the release process deviates substantially from a stationary binomial model, leading to large uncertainty in N. Because $p_{i}$ is computed directly from q, even moderate bias in N can propagate and produce amplified errors in the inferred release probability sequence.

To address this limitation, we introduced an additional calibration step based on the classical Tsodyks–Markram (T–M) short-term synaptic plasticity model. The T–M framework explicitly captures the dynamic evolution of synaptic efficacy during repetitive stimulation by incorporating two key presynaptic state variables: the fraction of available synaptic resources and the facilitation-dependent utilization of release.

In this model, the effective release probability at stimulus index i is expressed as

(20) $$p_{i}=U_{i}\cdot R_{i},$$

where $R_{i}$ denotes the fraction of readily releasable resources remaining before the i-th pulse, and $U_{i}$ represents the utilization parameter modulated by facilitation. Their dynamics follow

(21) $$\left\{\begin{array}{c} R_{i+1}=R_{i}(1-U_{i})+(1-R_{i}(1-U_{i}))e^{-\frac{∆t}{\tau_{d}}}\\ U_{i+1}=U_{i}e^{-\frac{∆t}{\tau_{d}}}+U_{0}(1-U_{i}e^{-\frac{∆t}{\tau_{f}}}) \end{array}\right.,$$

where $\tau_{d}$ and $\tau_{f}$ are the recovery and facilitation time constants, respectively, and ∆t is the inter-stimulus interval.

In practice, we first obtained initial moment-based estimates $\hat{q}$, $\hat{N}$, and $\hat{p_{i}}$ from Equations (16)–(18). The quantal size estimate $\hat{q}$ was highly stable across index combinations and therefore treated as fixed. We then fitted the T–M model to the experimentally observed mean EPSC trajectory $\mu_{i}$, using $\hat{q}$ as a scaling constraint, and optimized only the dynamic parameters $\left(U_{0},\tau_{d},\tau_{f}\right)$ together with a calibrated pool size $N_{cal}$.

This calibration is expected to regularize the vesicle pool estimate by enforcing physiologically consistent facilitation dynamics. By constraining the estimation process in this manner, the resulting $N_{cal}$ is anticipated to exhibit reduced dispersion and improved consistency with the underlying vesicle pool size in simulations. Consequently, the reconstructed release probability sequence $\hat{p_{i}}$ is expected to become smoother and more accurate, better reflecting the expected facilitation profile.

Together, this hybrid strategy combines the speed and analytical tractability of moment-based inference with the biophysical interpretability of the T–M model, enabling reliable estimation of both static synaptic parameters (q,N) and dynamic release probability modulation under short-term facilitation.

### 2.9. Ethical Approval Statement

The MATLAB codes in this article employs a hybrid programming approach combining manual and AI efforts. Although most of the repetitive plotting code was generated by AI, all data analysis and plotting code underwent manual inspection and modification

## 3. Results

### 3.1. Statistical Properties of EPSC Responses Under Stimulus Trains

To provide an intuitive understanding of the data structure underlying our method, we first examine how synaptic responses behave statistically under repeated stimulation. By recording excitatory postsynaptic currents (EPSCs) from CA1 PCs, we assessed the short-term synaptic facilitation evoked by a train of 10 stimuli at 20, 50, and 75 Hz to the presynaptic SC axons in acute hippocampal slices. In our results, repetitive stimulation at frequencies > 20 Hz elicited robust short-term facilitation of consecutive EPSCs. This short-term plasticity indicated elevated calcium ions at presynaptic release sites induced by stimulation. In our experiments, the relatively short inter-stimulus intervals resulted in nonlinear superposition of residual calcium ions from the preceding stimulus with newly influx calcium ions during subsequent stimulation. This interaction led to facilitated neurotransmitter release, manifesting as synaptic facilitation.

In synaptic transmission, inputs of varying frequencies can induce facilitation effects of distinct magnitudes, thereby enabling temporal filtering or amplification of signals. This frequency-dependent facilitation constitutes a fundamental mechanism underlying the response characteristics of neural circuits. The initial phase of a stimulation sequence establishes the basic trajectory and dynamic range for subsequent synaptic responses, with synapses exhibiting low initial release probability being more prone to short-term facilitation. Therefore, analyzing synaptic behavior during the initial stage of stimulation—particularly in the context of short-term plasticity—provides critical insights into the mechanisms of information processing within neural circuits over brief temporal windows.

To motivate the moment-based parameter estimation framework, we first examine the statistical properties of EPSC responses recorded under repetitive stimulus trains. Rather than focusing on model fitting at this stage, we aim to establish several robust empirical observations directly from the data, which provide the statistical foundation for the subsequent estimation procedures.

Across repeated sweeps within individual cells, EPSC amplitudes evoked by identical stimulus indices exhibit substantial trial-to-trial variability. Importantly, this variability is not purely attributable to baseline noise, but displays structured dependencies across stimulus indices, consistent with fluctuations in effective quantal size and release efficacy. These observations motivate the use of both first- and second-order moments to characterize synaptic transmission dynamics under short-term facilitation.

#### 3.1.1. Mean EPSC Dynamics During Short-Term Facilitation

We first show that synaptic responses follow a consistent and reproducible facilitation pattern across stimuli, which provides a stable foundation for parameter estimation. We first examined the evolution of mean EPSC amplitude across stimulus indices during short-term stimulus trains. For facilitating synapses, the mean EPSC typically increases over the initial stimuli before reaching a plateau or gradually declining, reflecting a balance between facilitation mechanisms and vesicle depletion.

**Figure 3:**
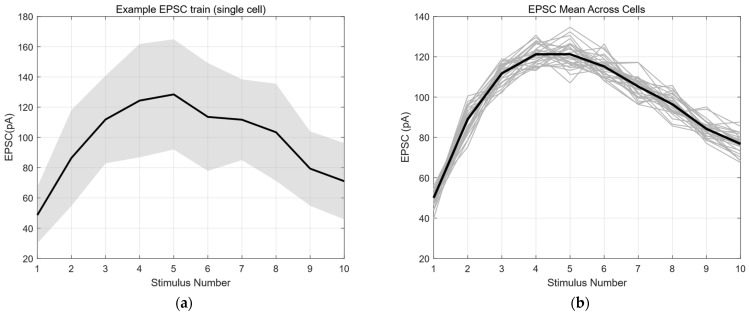

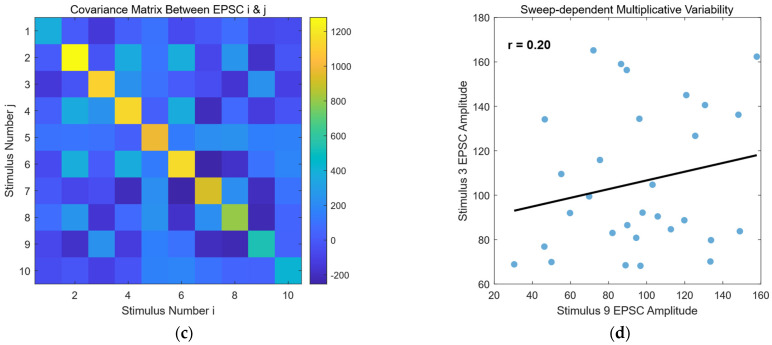
EPSC statistics underlying moment-based analysis. (**a**) An example EPSC amplitude sequence from a representative cell across repeated stimuli, illustrating stimulus-dependent modulation. Black line: mean EPSC peak amplitude across sweeps; gray shading: standard deviation. (**b**) Mean EPSC amplitude sequences across multiple cells of the same type, showing consistent stimulus-dependent profiles. The black trace indicates the pooled mean EPSC for all cells in this condition; individual gray traces represent the mean EPSC per cell (averaged over sweeps). (**c**) A heatmap of pairwise covariance between sweeps, predominantly positive, indicating a strong shared modulation consistent with release probability dynamics. (**d**) Representative pairwise sweep correlations, demonstrating modest variability consistent with small fluctuations in quantal size (q). Each dot represents the EPSC amplitude evoked by the i-th stimulus in two different sweeps. The black line indicates the linear regression fit to these data points.

Figure 3a shows the average EPSC amplitude as a function of stimulus number for a representative cell, computed across repeated sweeps. Despite variability at the single-trial level, the mean response exhibits a smooth, stimulus-dependent trajectory that is highly reproducible across cells. This observation supports the assumption that the stimulus-dependent release probability $p_{i}$ can be treated as a deterministic function of the stimulus index when considering ensemble statistics.

To assess consistency across the population, we normalized EPSC amplitudes by the first response and pooled data from multiple cells. As shown in Figure 3b, normalized mean EPSC trajectories display a stereotypical facilitation profile during the initial stimuli, with relatively small inter-cell dispersion. This population-level regularity justifies aggregating moment statistics across sweeps within each cell for parameter estimation.

#### 3.1.2. Second-Order Moment Structure of EPSC Responses

We next show that trial-to-trial variability is not random but structured across stimuli, revealing hidden dependencies that are not captured by mean responses alone. Beyond mean dynamics, we next investigated second-order statistical relationships between EPSC responses at different stimulus indices. Specifically, we analyzed the covariance structure of EPSC amplitudes across sweeps to determine whether trial-to-trial fluctuations are independent or correlated across stimuli.

Based on EPSC data obtained from repeated sweeps, we computed the pairwise covariance between EPSC amplitude vectors corresponding to different stimulus indices i and j, and visualized the results as a heatmap (Figure 3c). Specifically, assuming that 20 sweeps were recorded and each sweep contained 10 stimuli, the EPSC amplitudes corresponding to the i-th stimulus across all sweeps were extracted to form a vector. The covariance between this vector and that of the j-th stimulus was then calculated. The resulting covariance value was assigned to the (i,j)-th entry of the covariance matrix and visualized using a heatmap. Warmer colors (yellow) indicate higher covariance values, whereas cooler colors (dark blue) indicate lower covariance values. Notably, these covariances persist even for stimulus pairs that are temporally separated, indicating the presence of a shared sweep-level fluctuation component rather than purely local noise.

To further illustrate this effect, we examined scatter plots of EPSC amplitudes between selected stimulus pairs across sweeps (Figure 3d). These plots reveal elongated distributions along the diagonal, consistent with multiplicative trial-to-trial variability, rather than circular clouds expected from independent noise. Such structure directly supports the presence of sweep-dependent variability in quantal amplitude, a key assumption underlying the use of second-order moments in our estimation framework.

Importantly, the magnitude of covariance scaled approximately with the product of the corresponding mean EPSC amplitudes across stimulus pairs. This proportional relationship suggests that the second-order statistics are dominated by global fluctuations in effective quantal size rather than stimulus-specific noise sources, thereby enabling the elimination of unobservable variables through moment-based combinations in later sections.

Together, these results demonstrate that EPSC responses under stimulus trains exhibit structured first- and second-order statistical properties: smooth stimulus-dependent mean dynamics and robust cross-stimulus covariance driven by sweep-level fluctuations. These empirical observations validate the central premise of our approach—that second-order moments contain informative, non-redundant information about synaptic parameters—and motivate the moment-based elimination and estimation strategies developed in the following sections.

### 3.2. Estimation of Effective Parameters Using Moment-Based Elimination

Having established that EPSC responses to stimulus trains exhibit robust and structured first- and second-order statistical properties, we next demonstrate that these observable moments can be directly leveraged—via the proposed moment-based elimination strategy—to infer key synaptic parameters. Specifically, we applied the proposed moment-based elimination strategy to infer quantal size (q), effective vesicle pool size (N), and stimulus-dependent release probability ($p_{i}$) without requiring explicit fitting of the full stochastic release process.

All parameter estimates in this section were obtained using only empirically measured mean, variance, and covariance terms, following the elimination procedures described in the Methods Section. Importantly, no assumptions about the explicit functional form of facilitation dynamics were imposed.

#### 3.2.1. Quantal Size Estimation from Second-Order Moments

We first demonstrate that the quantal size q, the fundamental unit of synaptic response and a key scaling factor for EPSC amplitudes, can be reliably estimated using second-order statistics alone. Because our moment-based elimination strategy removes dependence on release probability and vesicle pool size through combinations of first- and second-order moments, q can be directly inferred from observable statistics across multiple stimulus indices, providing a foundation for subsequent parameter estimation.

**Figure 4:**
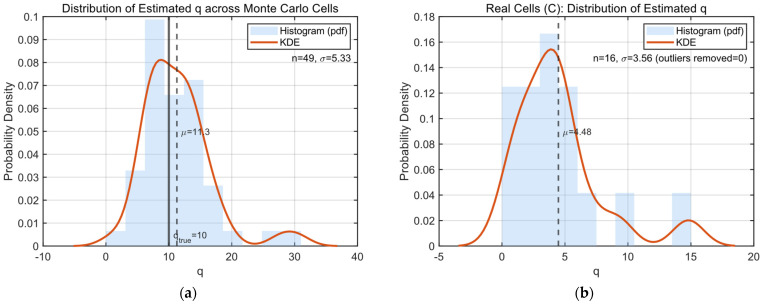

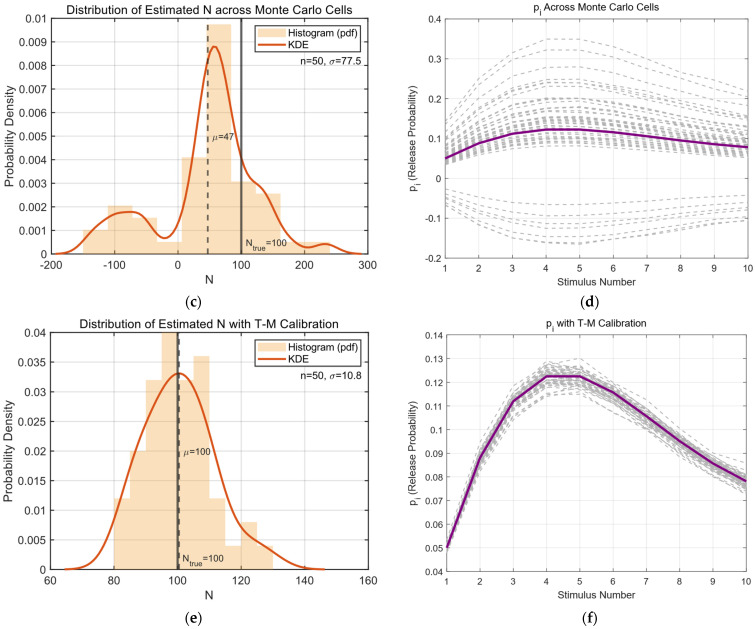
Estimation performance of synaptic parameters. Unless otherwise noted, cyan, orange, and purple are used to represent the results for parameter q, N, and release probability $p_{i}$, respectively. (**a**) Bootstrap distribution of the quantal size. KDE (Kernel Density Estimation) is a non-parametric method used to estimate the probability density function of a dataset. It works by placing a smooth kernel (typically a Gaussian function) at each data point and summing them to produce a continuous density curve, providing a clearer visualization of the underlying distribution compared to discrete histograms. The probability density curve shown in the figure is the KDE curve. (q) from a representative cell, showing tight convergence. (**b**) The distribution of q across cells of the same synaptic type, remaining within a physiologically reasonable range. (**c**) Estimated vesicle pool size (N), exhibiting larger dispersion compared with q. (**d**) The reconstructed release probability sequence ($p_{i}$) prior to calibration, showing increased variability across stimuli. The purple solid line represents the mean of the calculated release probabilities ($p_{i}$), while each gray dashed line depicts the release probability curve derived from the activity data of a single simulated neuron. (**e**) Calibrated $p_{i}$ dynamics, with a pronounced increase during the initial stimuli consistent with facilitation. (**f**) Calibrated $p_{i}$ trajectories across multiple cells, demonstrating improved cross-cell consistency.

Figure 4a illustrates the distribution of quantal size estimates obtained from different index combinations within a representative cell. Despite substantial trial-to-trial variability in raw EPSC amplitudes, the resulting q estimates cluster tightly around a well-defined central value. Occasional negative or extreme estimates, arising from noise-dominated index combinations, were rare and readily identifiable as outliers.

To assess consistency across cells, we pooled quantal size estimates from all recorded neurons (Figure 4b). Across the population, estimated q values fell within a narrow range and exhibited unimodal distributions, consistent with the notion that quantal size is a stable synaptic property within individual cells. These results demonstrate that second-order moment information alone is sufficient to robustly constrain quantal amplitude.

#### 3.2.2. Robustness of q, N, and $p_{i}$ Estimation Across Index Combinations

We then evaluate the stability and reliability of our estimation framework by analyzing the overall distribution of parameters derived from a large population of randomly generated simulated neurons. A key advantage of the moment-based elimination framework is its ability to compute parameter estimates efficiently across diverse neuronal configurations. Therefore, instead of relying on data subsets, we examined the robustness of the estimated parameters by assessing their statistical distribution over this extensive ensemble of synthetic neurons, which is critical for establishing practical applicability.

Figure 4c shows the distribution of estimated effective vesicle pool size N derived from different index pairs within the same cell. While raw estimates exhibit some dispersion, the majority fall within a biologically plausible range. Consistent with theoretical expectations, covariance-based estimates tend to slightly underestimate the true effective pool size; accordingly, we adopted a conservative post-processing strategy by selecting the maximum value within the central distribution range.

Similarly, the release probability estimates $p_{i}$ exhibited a clear stimulus dependence (Figure 4d). However, compared with the relatively stable estimates of q, the variability in $p_{i}$ across Monte Carlo cells was noticeably larger, and systematic deviations from the ground truth were observed. This increased dispersion can be primarily attributed to uncertainty in estimating the readily releasable pool size N. Although q can be reliably constrained by the elimination procedure, N is more susceptible to biological variability and sampling noise. Since $p_{i}$ is computed based on both q and N, inaccuracies or fluctuations in N propagate directly to the estimation of $p_{i}$, leading to amplified variability in the inferred release probabilities.

Despite these deviations, the overall stimulus-dependent trend was preserved, suggesting that the proposed framework captures the dominant dynamics of release modulation, while highlighting the sensitivity of $p_{i}$ estimation to uncertainties in pool size determination.

Together, these results confirm that the proposed estimation scheme is not overly sensitive to index selection and that averaging across combinations further enhances robustness.

#### 3.2.3. Improvement in N and $p_{i}$ Estimation After T–M Model Calibration

Finally, we show that incorporating a simple dynamical model can substantially improve the estimation of less stable parameters while preserving the accuracy of q.

Although the moment-based elimination framework provides reliable closed-form estimates of the quantal size q, the effective vesicle pool size N remains sensitive to deviations from the stationary binomial assumption, particularly under strong facilitation. As a result, variability in N directly affects the inferred release probability sequence $p_{i}$, leading to amplified variability in its stimulus-dependent profile (Figure 4c,d).

To mitigate this limitation, we applied the Tsodyks–Markram (T–M) model–based calibration described in Section 2.7, using the moment-derived q as a fixed scaling constraint and fitting only the dynamic parameters together with a calibrated pool size $N_{cal}$.

After calibration, the distribution of N estimates became markedly narrower and centered around physiologically plausible values (Figure 4e). Compared with the pre-calibration results, variance was substantially reduced and extreme or non-physical solutions were effectively suppressed.

Importantly, the refined pool size estimate led to a significant improvement in the reconstructed release probability trajectory. The calibrated $p_{i}$ sequence exhibited a smooth, monotonic facilitation profile with greatly reduced inter-cell dispersion (Figure 4f), closely matching the expected dynamics of short-term facilitation. In particular, the initial stimuli showed a pronounced increase in release probability, consistent with calcium-dependent facilitation mechanisms.

These results show that incorporating T–M dynamical constraints with moment-based inference substantially enhances the precision of both N and $p_{i}$ estimation. The hybrid strategy preserves the computational efficiency of the analytical framework while restoring biophysical consistency under facilitating synaptic conditions.

### 3.3. Comparison with Mean–Variance Analysis

**Figure 5:**
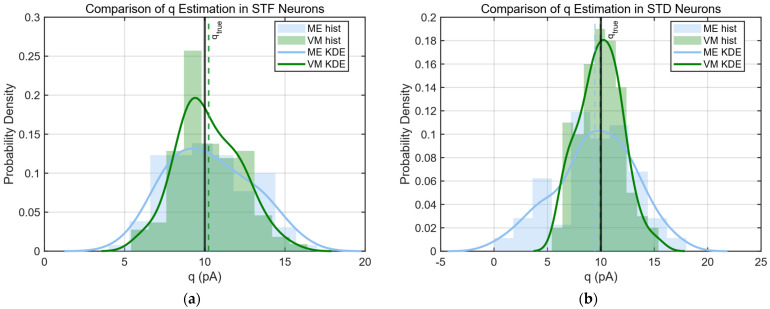

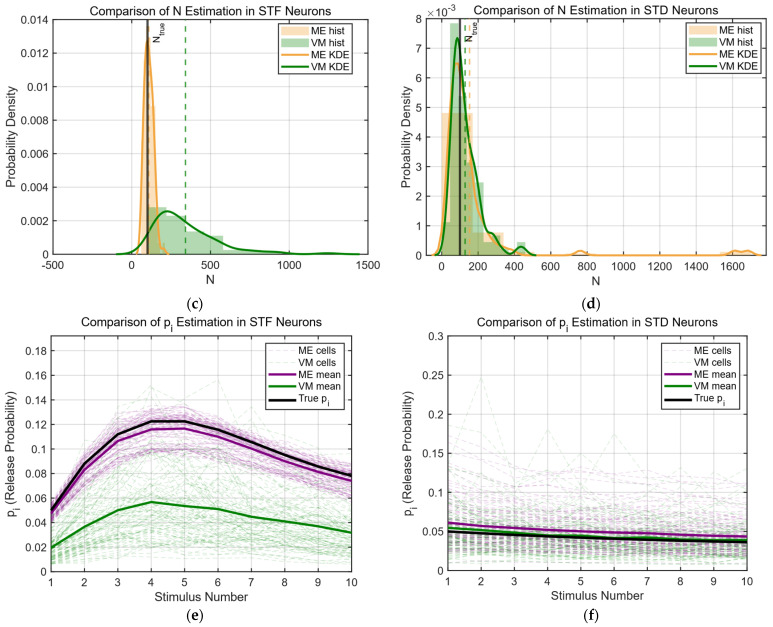
Comparison between the proposed moment-based (ME) parameter estimation framework and the classical mean–variance (VM) method. The left column (**a**,**c**,**e**) shows results obtained under simulated short-term facilitating (STF) synaptic conditions, while the right column (**b**,**d**,**f**) presents results under simulated short-term depressing (STD) conditions. As in Figure 4, the vertical dashed line indicates the mean of the distribution. Colors are consistent with the text: blue, orange, and purple represent q, N, and release probability $p_{i}$ for our proposed method, respectively, while green denotes the results of the VM algorithm. (**a**) Comparison of quantal size q estimation between the ME and VM methods under STF conditions. The green distribution corresponds to the VM estimates. (**b**) Same comparison as in (**a**), but under STD conditions. (**c**) Comparison of vesicle pool size N estimation between the two methods under STF conditions. (**d**) Comparison of N estimation under STD conditions. (**e**) Comparison of release probability $p_{i}$ estimation under STF conditions. (**f**) Comparison of $p_{i}$ estimation under STD conditions.

To further evaluate the performance of the proposed moment-based estimation (ME) framework, we compared it with the classical mean–variance (VM) method under both short-term facilitating (STF) and short-term depressing (STD) synaptic conditions (Figure 5a–f).

Under STF conditions (Figure 5a,c,e), the ME method demonstrated substantially improved accuracy and stability compared with the VM approach. In particular, the estimated quantal size q showed a tighter distribution centered around the ground truth, whereas VM estimates exhibited broader dispersion. More strikingly, the estimation of vesicle pool size N by the VM method was highly unstable, with large variance and severe overestimation (Figure 5c), consistent with the quantitative results summarized in Table 1, where the RMSE of N under VM (STF) was an order of magnitude larger than that of the ME method. Similarly, the reconstructed release probability $p_{i}$ using the ME framework closely followed the true facilitation trajectory, while the VM method systematically underestimated $p_{i}$ and failed to capture its dynamic increase across stimuli (Figure 5e).

In contrast, under STD conditions (Figure 5b,d,f), the performance of the two methods became more comparable. The VM approach showed slightly lower bias and RMSE in the estimation of q and N, consistent with its strong underlying assumptions tailored to depressing synapses. The ME method, while exhibiting a modest reduction in estimation precision in this regime, still produced stable and physiologically plausible parameter estimates. This slight loss in accuracy reflects a deliberate trade-off in the ME framework, where part of the constraint on release probability is relaxed to improve robustness and applicability across more general synaptic dynamics.

The observed discrepancy between the two methods can be attributed to their underlying statistical assumptions. The VM method relies on a parabolic relationship between mean and variance derived from a stationary binomial model, which implicitly assumes independent release events and often depends on negative covariance or variance reduction across successive stimuli. Such assumptions are well satisfied in STD synapses, where vesicle depletion leads to reduced release probability and decorrelated responses.

However, in STF synapses, presynaptic calcium accumulation introduces strong, stimulus-dependent increases in release probability, along with structured, positive cross-stimulus covariance in EPSC amplitudes. As demonstrated in earlier sections, these covariance structures reflect shared sweep-level fluctuations rather than independent binomial variability. Because the VM framework does not incorporate cross-stimulus covariance, it fails to account for this structured variability, leading to biased and unstable parameter estimates under facilitation.

In contrast, the proposed ME method explicitly incorporates second-order cross-stimulus covariance and leverages it to eliminate latent variables such as $p_{i}$. This allows the method to capture the non-stationary and correlated nature of synaptic transmission in STF regimes. As a result, it achieves significantly improved robustness and accuracy when facilitation dominates, while maintaining competitive performance in STD conditions.

Overall, these results suggest that the ME framework provides a more flexible estimation strategy that extends beyond the strict assumptions of classical VM analysis. By partially relaxing constraints on release probability and incorporating cross-stimulus covariance, the method achieves improved robustness to noise and broader compatibility with dynamic synaptic regimes such as STF. This gain in generality is accompanied by a minor reduction in estimation precision under conditions where the VM assumptions are well satisfied (e.g., STD synapses). Nevertheless, the ME approach maintains consistent and reliable performance across both facilitating and depressing regimes, highlighting its practical value as a unified framework for synaptic parameter inference.

### 3.4. Sensitivity and Noise Robustness Analysis

To evaluate the practical applicability of the proposed moment-based elimination scheme, we systematically examined its sensitivity to measurement noise, quantal variability, and sampling size. Because the estimator explicitly incorporates second-order statistics and eliminates nuisance parameters analytically, its stability under realistic experimental perturbations is a key determinant of performance.

We assessed robustness along three major dimensions:

- Measurement noise level $\sigma_{\eta }^{2}$.
- Quantal variability $\sigma_{q}^{2}$ (or equivalently $R_{i,j}=\frac{\sigma_{q}^{2}}{q^{2}}$);
- Sweep number T.

Both simulated datasets (with known ground-truth parameters) and representative experimental datasets were used for validation.

#### 3.4.1. Sensitivity to Measurement Noise $\sigma_{\eta }^{2}$

We first tested whether the method remains accurate in the presence of measurement noise, which is unavoidable in electrophysiological recordings. Measurement noise enters the EPSC model additively as Gaussian noise $\eta_{i}\sim Normal(0,\sigma_{\eta }^{2})$. In the derivation of $F_{i,j}$, the term $\sigma_{\eta }^{2}$ is explicitly subtracted, theoretically eliminating its biasing effect.

To test the effectiveness of this correction, we simulated datasets with increasing noise variance while keeping q, N, and $p_{i}$ fixed. Noise levels were scaled relative to mean EPSC amplitude (e.g., 0–50% of peak current).

The results showed the following:

1. The estimator of q remained unbiased across a wide range of noise levels.
2. Variance in estimates increased gradually but did not diverge.
3. N estimates were slightly more sensitive but remained stable within physiologically relevant noise ranges (referring to when the noise standard deviation $\sigma_{\eta }$ was less than 30% of the EPSC mean). However, it should be noted that when the $\sigma_{\eta }\geq 30\%$ of the average EPSC value, both the N value calculated directly using moment information and the calibrated N value obtained through fitting with the T-M model will exhibit significant deviations.
4. Traditional mean–variance methods exhibited strong bias inflation at moderate noise levels.

**Figure 6:**
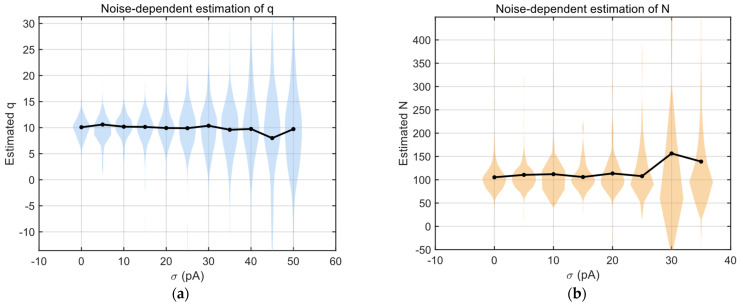

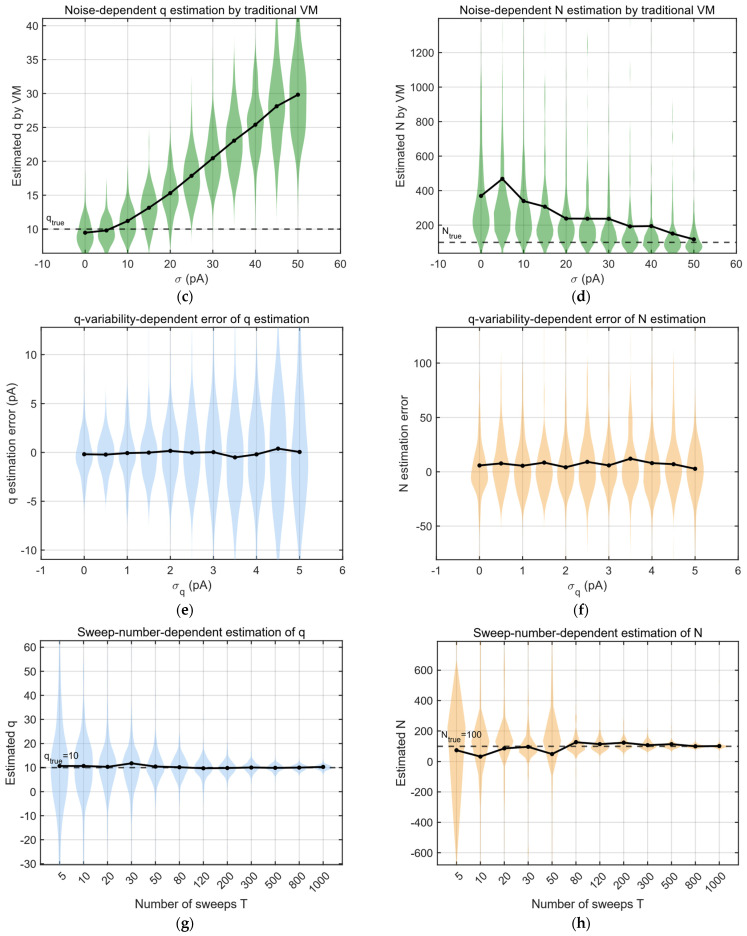

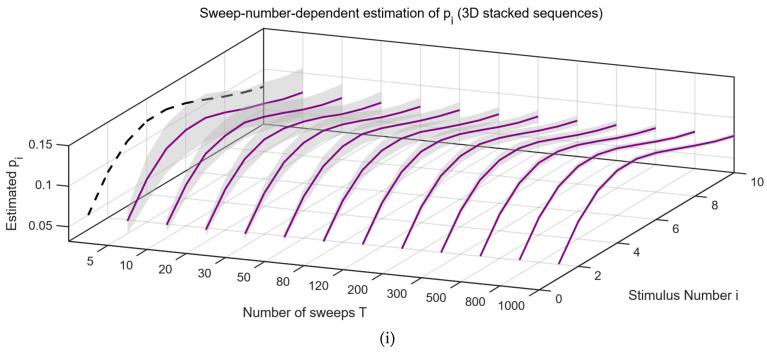
Overall analysis and calculation of the scheme’s resistance to noise. In panels a–h, all dots represent the mean of the distribution. The solid black line traces the changes in the mean of the parameter estimates across varying simulation conditions, while the shaded area indicates the distribution. (**a**) As the random noise gradually increases, the error in the estimated value of q will also gradually increase. However, the overall property of unbiased estimation is still maintained. (**b**) As the random noise gradually increases, the error in estimating N also gradually increases, but it remains controllable. However, when the standard deviation of the random noise exceeds 40% of the EPSC mean, a fitting disaster occurs, and a large number of negative numbers or other impossible situations appear in the subsequent solutions for N. (**c**) Estimation of the quantal size q using the VM method under progressively increasing random noise. As the noise level increases, the variability in the estimates grows as expected; in addition, a systematic upward bias in the estimated mean of q becomes evident. (**d**) Estimation of the vesicle pool size N using the VM method under increasing random noise. Interestingly, a counterintuitive trend is observed: as the noise level increases, the estimated N gradually approaches the ground-truth value. (**e**) As the random variation in q between sweeps gradually increases, the estimation error of q also becomes larger. Here, the vertical axis has been replaced because the true value of q changes every time. Therefore, in order to better demonstrate the calculation effect, we have replaced the absolute value of q with the error between the estimated q and the current true q. The error can be positive or negative, but the overall mean remains near 0, still providing an unbiased estimation and exhibiting good resistance to q variation. (**f**) As the random variation of q between sweeps gradually increases, the estimation error of N remains stable. (**g**) As the number of iterations T of the sweep increases, the estimation of q becomes increasingly accurate. When T reaches around 20 to 30, the estimation of q is already basically usable and can effectively perform the statistical inference required for experiments, identifying differences of around 10pA (3σ≈10pA). (**h**) Similarly, as T increases, the estimation of N becomes more accurate. However, since N is more sensitive to random variation, the estimation results may vary greatly. If precise analysis of N is desired, it is recommended to sweep to 80 or above. Of course, if the number of cells collected during use is sufficient, the sweep count can be appropriately reduced to the conventional sample size range of 20 to 30. (**i**) As T increases, the estimation of the release probability $p_{i}$ gradually converges, and we can observe that the estimation of $p_{i}$ is also an unbiased estimation. The black dashed line on the far left indicates the true value of the release probability used in the simulation setup. Other visual elements follow the same scheme as the release probability curves described earlier.

These results indicate that explicit noise elimination in $F_{i,j}$ effectively prevents systematic distortion under realistic recording conditions (Figure 6a,b).

#### 3.4.2. Robustness to Quantal Variability

We then assess whether the method can handle biological variability in quantal size across trials. Unlike rapid approximation methods assuming $\sigma_{q}^{2}\to 0$, the detailed computation branch explicitly estimates the ratio $R_{i,j}=\frac{\sigma_{q}^{2}}{q^{2}}$ via covariance normalization.

We systematically varied $\frac{\sigma_{q}}{q}$ from 0 to 0.5 in simulations to assess estimator stability.

The findings are as follows:

1. A detailed branch incorporating $\hat{R}$ remains unbiased across the tested range.
2. Estimation variance increases mildly but remains bounded.
3. Error scales approximately linearly with $\hat{R}$, not exponentially.

This confirms that covariance-normalized correction successfully compensates for sweep-level quantal fluctuation (Figure 6e,f).

#### 3.4.3. Sensitivity to Sweep Number T

Finally, we evaluate how much data are required for reliable estimation, which is critical for experimental feasibility. Moment-based estimators rely on empirical estimates of first- and second-order statistics (mean, variance, and cross-stimulus covariance), all of which are computed across repeated sweeps. Therefore, the number of available sweeps T directly determines the sampling accuracy of these moment estimates, and consequently affects the stability of the inferred synaptic parameters.

To evaluate the dependence of estimation performance on T, we performed Monte Carlo simulations with fixed ground-truth parameters $\left(q,N,p_{i}\right)$, while systematically varying the number of sweeps used for moment computation. For each T, we generated multiple synthetic datasets and applied the full estimation pipeline described in Section 2.6, including outlier rejection and averaging across valid index tuples.

The results showed the following:

1. q estimation rapidly converged with increasing T. Even with relatively small sweep numbers, the estimator remained nearly unbiased, and the dispersion decreased monotonically as T increased (Figure 6g). This behavior is expected because the closed-form expression for q is dominated by ratios of moments (e.g., $F_{i,j}$ and $\mu_{i}$), which tend to converge quickly under repeated sampling.
2. The variance of q estimates decreased approximately as a function of $\frac{1}{\sqrt{T}}$. This scaling is consistent with standard statistical convergence properties of sample mean and covariance estimators. Importantly, no systematic drift was observed in the mean estimated *q* across tested sweep numbers, indicating that limited *T* primarily introduces variance rather than bias.
3. N and *pi* were substantially more sensitive to small T. In contrast to q, estimation of *N* depends critically on second-order terms that involve variance subtraction and binomial structure (Figure 6h,i). Under small T, sampling fluctuations in $Var(A_{i})$ and $Cov(A_{i},A_{j})$ can produce unstable or non-physical intermediate values (e.g., negative candidate N). Because $p_{i}$ is computed directly from q and N, such instability propagates and amplifies variability in reconstructed release probability sequences.
4. A practical sweep threshold exists for stable inference. In our simulation settings, when T was sufficiently large, the inferred q distributions became narrow and reproducible across repetitions, while N estimates remained within physiologically plausible ranges after post-processing. However, when T dropped below a critical range, the proportion of outlier solutions increased markedly, and dispersion of N and $p_{i}$ became difficult to control.

These results demonstrate that the proposed moment-based scheme is highly sample-efficient for estimating the quantal unit q, but requires a moderate number of sweeps to stabilize pool-size-related quantities. In practice, this is not a major limitation, since typical electrophysiological recordings routinely include 15–30 sweeps per condition. Overall, the sweep number analysis confirms that the primary strength of our method—robust recovery of q—remains reliable even under limited sampling, while N and $p_{i}$ can be further stabilized using the T–M calibration step described in Section 2.7.

#### 3.4.4. Parameter Calculation Under Non-Ideal Conditions

We further test the robustness of the method under biologically realistic heterogeneity across synaptic release sites. Synaptic transmission is inherently heterogeneous across release sites within a single synapse. This heterogeneity arises from multiple well-established biophysical factors. First, the quantal amplitude q can vary across sites due to differences in vesicle size, neurotransmitter content, and postsynaptic receptor density or subunit composition. Variability in receptor clustering and nanodomain organization at the postsynaptic density further contributes to site-specific differences in postsynaptic response amplitude. Second, the release probability p is strongly influenced by the spatial coupling between synaptic vesicles and voltage-gated calcium channels. Differences in the nanoscale distance to calcium sources, as well as local variations in calcium buffering and channel density, lead to systematic offsets in release probability across sites. Importantly, these two sources of heterogeneity—quantal size and release probability—are generally governed by distinct molecular and structural mechanisms and are therefore expected to be largely independent.

In addition, short-term plasticity mechanisms, such as facilitation and depression, can further amplify these differences across repeated stimulation. Variability in vesicle priming states, replenishment kinetics, and calcium sensitivity introduces site-dependent dynamics in release probability over time. As a result, even within a single synapse, the effective parameters governing neurotransmission are distributed rather than uniform, making it critical to assess how such heterogeneity affects parameter estimation methods.

**Figure 7:**
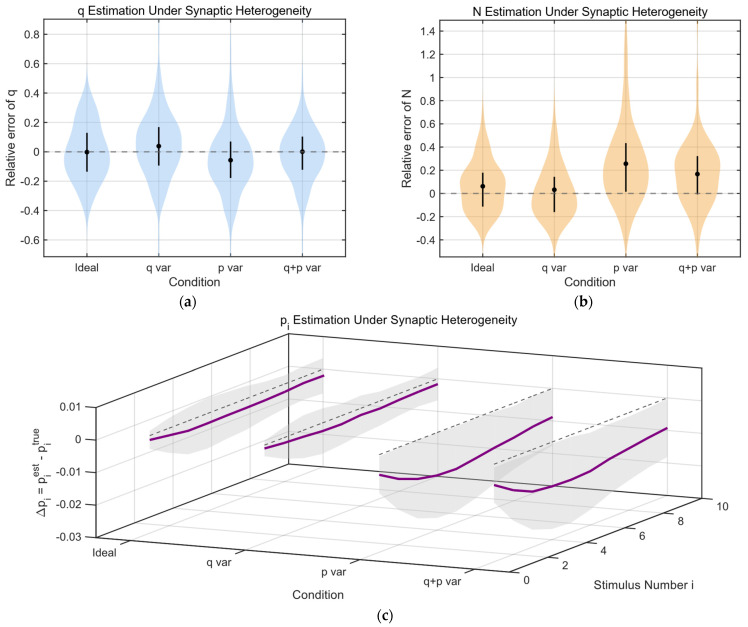
The robustness of parameter estimation under synaptic heterogeneity. In contrast to the preceding figures, this figure shows relative errors only. The dashed line indicates the ground truth. (**a**) The relative error of quantal size q estimation under four conditions: ideal, q variability, p variability, and combined heterogeneity. Variability in q leads to slight overestimation, whereas variability in $p_{i}$ induces mild underestimation. Black dots: mean error; black vertical lines: one standard deviation. (**b**) The relative error of release site number N estimation. q variability has minimal effect, while p variability causes pronounced overestimation, which persists under combined heterogeneity. (**c**) The estimation error of release probability $p_{i}$ across stimuli (mean ± s.d.). Variability in q has negligible impact, whereas variability in p leads to systematic underestimation. Purple solid line, mean error of release probability estimates; gray shaded area, one standard deviation.

To evaluate the robustness of our estimation framework under biologically realistic heterogeneity, we systematically introduced site-to-site variability in both q and p. In the simulation conditions, in order to more clearly see the impact of heterogeneity, we provided larger standard deviations of inter site heterogeneity, with $\sigma_{q}=2$ and $\sigma_{p}=0.05$. Our simulations reveal distinct and interpretable biases in the estimated parameter (Figure 7a). When heterogeneity is present only in quantal amplitude, the estimated q exhibits a slight upward bias, consistent with the fact that the estimator effectively depends on second-order moments and is therefore sensitive to the variance of q across sites. In contrast, heterogeneity in release probability leads to a mild downward bias in the estimated q, reflecting a reduction in the effective binomial variance component. When both sources of heterogeneity are present, these effects approximately superimpose, resulting in a net bias that reflects the balance between the two mechanisms.

For the estimation of the number of release sites N, we observe that variability in q alone has minimal impact, indicating that the estimation of N is relatively robust to quantal amplitude heterogeneity. However, heterogeneity in release probability produces a pronounced overestimation of N. This effect arises because site-dependent offsets in p reduce the variance attributable to stochastic release, which the model compensates for by attributing the reduced variability to a larger number of release sites. When both q and p heterogeneity are present, the bias in N remains dominated by the contribution from p variability (Figure 7b).

Finally, as shown in Figure 7c, for the estimated release probability sequence $p_{i}$, we find that heterogeneity in q has negligible influence, as its effects are largely absorbed by the coupled estimation of q and N. In contrast, heterogeneity in p consistently leads to an underestimation of release probability, particularly in the low-probability regime relevant to facilitating synapses. This bias reflects the nonlinear relationship between mean response and underlying release probability in the presence of site-dependent offsets. Together, these results demonstrate that while the proposed method remains broadly robust, site-to-site variability in release probability represents the primary source of systematic bias in parameter estimation.

## 4. Discussion

### 4.1. Summary of Principal Findings

We developed a fast framework for estimating synaptic parameters based on first- and second-order moments of EPSC responses during short-term facilitation. By analytically eliminating latent variables through covariance–mean combinations, we derived closed-form estimators for quantal size q, effective vesicle pool size N, and stimulus-dependent release probability $p_{i}$.

The main findings are as follows:

1. Second-order cross-stimulus covariance contains structured, non-redundant information about sweep-level variability.
2. The quantal size q can be robustly and nearly unbiasedly estimated from moment-based elimination.
3. The effective pool size N is intrinsically unstable under a purely binomial assumption when facilitation dynamics are strong.
4. Release probability $p_{i}$ inherits uncertainty from N, leading to amplified dispersion.
5. Introducing a Tsodyks–Markram (T-M)-based calibration step regularizes N and restores physiologically consistent release probability dynamics.

Together, these results establish a hybrid framework that combines analytical tractability with biologically constrained dynamical calibration.

### 4.2. Why Second-Order Moments Are Essential

Traditional mean–variance (VM) approaches estimate synaptic parameters by exploiting the parabolic relationship between variance and mean under binomial assumptions [11]. However, such approaches treat individual stimuli as statistically independent and therefore neglect structured sweep-level variability across stimulus indices.

Our empirical results demonstrate consistent positive covariance between EPSC amplitudes evoked by different stimuli within the same train. Importantly, this covariance scales approximately with the product of their mean amplitudes, indicating multiplicative sweep-level fluctuations rather than independent additive noise.

This observation implies the following:

- Short-term plasticity cannot be decomposed into independent binomial draws per stimulus.
- Sweep-level quantal fluctuations constitute a shared latent factor.
- Cross-stimulus covariance carries eliminable information about $N^{2}\cdot p_{i}^{2}$ terms.

By incorporating covariance explicitly, our framework captures information that is invisible to classical VM methods. This represents a conceptual shift: second-order statistics are not merely noise descriptors, but structured carriers of mechanistic information.

### 4.3. Robust Estimation of Quantal Size

Among all inferred parameters, quantal size q exhibited the highest stability across index combinations, noise conditions, and quantal variability levels.

This robustness arises from two factors:

1. Analytical elimination removes quadratic $N^{2}\cdot p_{i}^{2}$ contributions.
2. The introduction of co-variance effectively resists sweep-level quantal variability.

Even under moderate measurement noise (<30% of mean EPSC amplitude), q remained effectively unbiased. In contrast, traditional VM approaches show rapid bias inflation under similar noise conditions [12,20], as shown in Figure 6c.

Because q serves as a global scaling factor linking synaptic currents to vesicle release events, its accurate recovery is critical. The stability of q therefore represents the primary strength of the present method.

### 4.4. Intrinsic Limitations of Binomial Pool Size Estimation

In contrast to q, the effective vesicle pool size N displayed substantial dispersion under the pure binomial framework. This instability is not merely numerical but conceptual.

The classical binomial model assumes the following: a fixed pool size, and stationary release probability per stimulus. These assumptions derive from the original quantal hypothesis and binomial formulations of transmitter release [21,22].

However, during short-term facilitation, release probability evolves dynamically due to presynaptic calcium accumulation [1]. Under such conditions, N becomes an effective statistical parameter rather than a direct anatomical quantity.

Furthermore, the estimation of N critically depends on the underlying variance structure, which is highly sensitive to measurement noise. Errors in estimating N propagate multiplicatively into the inferred release probabilities $p_{i}$. Consequently, our findings underscore a fundamental limitation: static binomial inference frameworks are inherently insufficient to fully capture synaptic dynamics under facilitation regimes.

### 4.5. Noise Robustness and Practical Applicability

Our sensitivity analyses indicate the following:

- q remains unbiased across realistic patch clamp noise levels.
- N remains stable when noise < 30% of the mean EPSC amplitude.
- Above ~40%, both direct and calibrated methods deteriorate.

Given that typical whole-cell recordings under controlled conditions exhibit substantially lower relative noise, the method is practical for standard in vitro electrophysiology.

Additionally, explicit subtraction of measurement noise in the composite observable $F_{i,j}$ prevents systematic distortion, a common weakness of classical VM methods, as shown in Figure 6c,d.

Another intriguing observation is that, as the level of random noise gradually increases, the distribution of the parameter N estimated by using the VM method exhibits progressive sharpening at Figure 6d—a trend contrary to conventional intuition. Upon closer examination, this phenomenon can be attributed to the fact that both the VM method and the approach proposed in this study first estimate the quantal size q, and subsequently derive N (the number of release sites) and p (the release probability) based on q together with constraints imposed by the mean and variance of experimentally measured EPSCs. Consequently, as the estimated q systematically increases with rising noise levels, the resulting estimate of N is correspondingly driven downward to satisfy the fixed moment constraints, thereby producing an apparently narrower distribution.

### 4.6. Practical Considerations on Stimulus Train Length and Experimental Design

In addition to the number of sweeps and noise level, the length of the stimulus train is another important factor affecting the applicability and stability of the proposed covariance-based estimator. In our formulation, at least four stimuli are required to compute the estimator of q, as the closed-form solution relies on multiple cross-stimulus moment relationships. A minimum number of distinct stimulus indices is necessary to eliminate stimulus-dependent release probabilities and obtain a valid solution. Consequently, paired-pulse protocols (two stimuli) and even three-stimulus paradigms are insufficient for applying the proposed method.

Importantly, this minimum requirement for computability should not be confused with the requirement for stable and unbiased estimation. When only four stimuli are used, the number of admissible index combinations remains limited, which reduces the effectiveness of averaging and outlier rejection. In practice, estimation stability improves as the number of stimuli increases, since additional stimuli provide more independent moment relationships and thus enhance statistical robustness.

Nevertheless, it is difficult to prescribe a universal minimum stimulus count for stable estimation, as the optimal train length depends strongly on experimental conditions, including synapse type, release probability dynamics, recording noise, and preparation quality. Instead of providing a fixed recommendation, we suggest that the stimulus train should be designed to capture the dynamic phases of synaptic response, where EPSCs exhibit substantial changes across stimuli. Once the response approaches a steady state, additional stimuli tend to contribute limited new information for parameter estimation.

In our experiments, we adopted trains of 10 stimuli, which reliably capture both the facilitating and subsequent depressive phases of synaptic transmission under stimulation frequencies of 20–75 Hz. This regime provides a rich set of informative dynamics for parameter estimation while maintaining experimental feasibility.

To further assist experimental design under varying conditions, we provide an open-source simulation framework in our GitHub repository https://github.com/StandardExpert/A-Fast-Synaptic-Parameter-Estimation-Method (accessed 26 March 2026). This tool allows users to generate synthetic synaptic responses based on user-specified parameters and experimental constraints, enabling them to estimate the required stimulus train length, number of sweeps, and sample size needed to achieve stable q recovery in their specific settings.

### 4.7. Functional Implications of Short-Term Plasticity Quantification

Short-term synaptic plasticity (STP) plays a central role in shaping synaptic information processing and circuit dynamics. In mammalian brain networks, synapses with low initial release probability typically exhibit facilitation and function as high-pass filters, preferentially transmitting high-frequency bursts. In contrast, synapses with high initial release probability often undergo short-term depression and act as low-pass filters, reliably transmitting low-frequency inputs while attenuating sustained high-frequency activity [3,6]. Importantly, neuromodulators can dynamically regulate presynaptic release probability, thereby shifting synaptic filtering properties and altering frequency selectivity [5].

At the network level, STP contributes to competitive interactions among neuronal populations. By modulating presynaptic release probability and vesicle replenishment rates, STP influences the balance between excitatory and inhibitory activity and supports winner-take-all-like dynamics. These mechanisms are particularly relevant for computational functions such as pattern separation and pattern completion in hippocampal circuits [23,24].

Disruptions of STP have been implicated in neuropsychiatric disorders, including schizophrenia and autism spectrum disorders [25,26]. Even when single-neuron firing properties remain relatively intact, abnormal short-term dynamics can impair population-level temporal coordination, such as theta–gamma synchronization, leading to deficits in cognitive flexibility and memory performance.

Finally, STP can be viewed as a dynamic substrate for process memory. By transiently redistributing synaptic efficacy, it enables circuits to adapt information processing on short timescales. Interactions between long-term plasticity and STP further modulate these dynamics, highlighting the importance of quantitatively characterizing short-term synaptic parameters.

Collectively, these functional considerations underscore that precise estimation of release probability and vesicle dynamics provides a critical link between presynaptic mechanisms, circuit computation, and behavior.

### 4.8. Future Directions

The present hybrid framework combines moment-based analytical inference with physiologically constrained dynamical calibration. Although validated here under facilitating synaptic conditions, several extensions warrant future investigation.

First, while stimulus-dependent release probability is constrained using a phenomenological model, direct integration of presynaptic calcium dynamics may further improve mechanistic interpretability. Incorporating calcium imaging or calcium-dependent release models could help disentangle vesicle availability from calcium-triggered release efficacy [1,27].

Second, the analytical and computational efficiency of the method makes it suitable for application to larger datasets, including in vivo recordings or high-throughput electrophysiological paradigms. With appropriate noise handling and non-stationarity correction, the framework may enable scalable quantification of short-term plasticity across cell populations.

Moreover, the present framework is closed-form and computationally efficient, making it well suited as an initialization step for more computationally intensive inference methods. In particular, the moment-based estimates $\hat{q},\;\hat{N},\;and\;\hat{p_{i}}$, derived directly from first- and second-order statistics, can be used as starting values for Bayesian or Monte Carlo-based approaches (e.g., MCMC or sequential Monte Carlo), or as prior mean parameters in probabilistic formulations. Because these estimates are already constrained by observable statistical structure, they define a biologically plausible region of parameter space and can help reduce sensitivity to initialization and convergence to suboptimal local solutions. We note that such integration has not been systematically implemented or benchmarked in the present study and remains an important direction for future work.

Finally, because short-term plasticity abnormalities are implicated in several neuropsychiatric and neurological disorders [25,26], the present approach may serve as a quantitative tool for characterizing disease-associated presynaptic dysfunction. Future work may explore its utility in pharmacological or disease models to systematically profile alterations in quantal size and release dynamics.

In summary, the proposed framework provides a flexible foundation for extending moment-based synaptic inference toward broader physiological, computational, and translational applications.

## 5. Conclusions

In this study, we developed a fast synaptic parameter estimation framework tailored for short-term facilitating synapses. By analytically combining first- and second-order statistical moments, the proposed method eliminates latent variables and yields closed-form estimators for quantal size, effective vesicle pool size, and stimulus-dependent release probability. The quantal size can be robustly and nearly unbiasedly recovered even under realistic levels of measurement noise and quantal variability. Although pool size estimation is intrinsically sensitive under dynamic facilitation, integration with Tsodyks–Markram model calibration restores physiological consistency and improves stability.

Overall, this hybrid moment-based approach provides a computationally efficient and biologically grounded tool for quantifying presynaptic dynamics, offering practical value for studying synaptic computation and characterizing presynaptic dysfunction in neurological and neuropsychiatric disorders.

## Figures and Tables

**Table 1:** Parameter estimation error of two methods.

Method	$q_{bias}$	$q_{RMSE}$	$N_{bias}$	$N_{RMSE}$	$p_{bias}$	$p_{RMSE}$
ME(STF)	0.2671	2.5600	8.3234	28.2980	−0.0050	0.0105
VM(STF)	0.2362	1.9688	241.1238	312.6750	−0.0553	0.0610
ME(STD)	−0.5575	3.7219	52.9756	240.4571	0.0084	0.0264
VM(STD)	−0.0485	2.0017	28.6086	81.5496	0.0025	0.0260

## Data Availability

All data and codes will be shared on Github later at https://github.com/StandardExpert/A-Fast-Synaptic-Parameter-Estimation-Method. The program is written in MATLAB 2024b.

## Supplementary Materials

The following supporting information can be downloaded at https://www.mdpi.com/article/10.3390/biomedicines14040771/s1, Section S1: Overall Rational, Table S1: Definitions of Symbols, Section S2: Calculation Process, Section S3: Error Analysis under Non-Ideal Neuronal Conditions, Section S4: Error Analysis under Non Gaussian Noise Conditions.
