# Supplementary material for: A Fast Synaptic Parameter Estimation Method Based on First- and Second-Order Moments for Short-Term Facilitating Synapses
Source: Biomedicines. 2026 Mar 28;14(4):771. doi: 10.3390/biomedicines14040771 (PMC13113672; doi:10.3390/biomedicines14040771)
Supplement: Supplementary file 1 [file biomedicines-14-00771-s001.zip › biomedicines-4186671-supplementary.pdf]

biomedicines

MDPI

Complete formula derivation process

Section S1 Overall Rationale.

To estimate synapse-related dynamical parameters in a way that is applicable to both depressing and facilitating synapses, we integrate the modeling frameworks of two prior studies and propose a fast computation scheme based on second-order moments.

Because the full synaptic activity dynamics constitute a complex stochastic differential equation that is difficult to solve directly, we simplify the computation by using covariances to eliminate the need for explicit calculation of PPP. In addition, we employ maximum-likelihood estimation to jointly exploit most of the moment information contained in the data, thereby improving the robustness and reliability of the inferred results.

Table S1. Definitions of Symbols

Symbols	Description
i, j, k, l	Represents the stimulus number in the stim train. There are multiple numbers used to represent several numbers, and depending on the specific situation, several numbers may be the same or different. Please pay attention to the explanation after reviewing the equation.
A_i	The amplitude of EPSC Amplitude current obtained after the i-th electrical stimulation, measured in picoamperes (pA). If the experimental data is EPSP, it is also applicable, but it will change the dimensions of subsequent related parameters.
k_i	The equivalent vesicle release number follows a binomial distribution $k_i \sim Binomial(N, p_i)$. $E(k_i) = N \cdot p_i$.
N	Effective size of vesicle pool. Note that due to the large total vesicle pool of facilitation type neurons, we cannot calculate other inactive vesicle pool parts that are on standby until their discharge activity reaches its maximum. Therefore, the calculated N is essentially the effective N_{effect}, which will be smaller than the true N_{total}.
p_i	The release probability of vesicle release sites involved in the work.
q_{sweep}	The random variation of a quantum unit within a sweep follows $q_{sweep} \sim Normal(q, \sigma_q^2)$.
T	Repeatedly measured sweep numbers.
q	The expected value of a quantal unit in a certain cell.
η_i	Random noise, following $\eta_i \sim Normal(0, \sigma_\eta^2)$. Which can be interpreted as resting-state noise in the data.
$Var(A_i)$	The variance of the EPSC random variable.
C	The covariance of the EPSC random variable.

 https://doi.org/10.3390/xxxxx

μ_i	The mean EPSC of a cell triggered after the i-th stimulus is taken from multiple repeated experiments.
$F_{i,j}$	The combination term that can obtain observation values is convenient for simplifying formula derivation. The specific definition is: $F_{i,j} = Var(A_i) - \frac{\mu_i}{\mu_j} Cov(A_i, A_j) - \sigma_\eta^2$
$R_{i,j}$	Representing the proportional relationship between σ_q^2 and q^2, facilitating simplified writing during formula derivation. $R_{i,j} = \frac{\sigma_q^2}{q^2} = \frac{Cov(A_i, A_j)}{\mu_i \cdot \mu_j}$

Section S2 Calculation Process

EPSC Definition

We assume that the amplitude of the postsynaptic current (EPSC) under each stimulus is a random variable, denoted by A_i (in pA).

$$A_i = q_{sweep} \cdot k_i + \eta_i$$

Here, q_{sweep} represents the quantal unit that varies across experimental repeats (sweeps), and the variation within a single sweep is assumed to be negligible.

k_i is the number of synaptic vesicles released after electrical stimulation under the i-th stim train condition. Based on the classical binomial distribution model, the stimulated release result of vesicles at each release site can only be 0 or 1, and each synaptic terminal has N independent release sites. $Var(k_i)$ is the distribution variance. The probability of release after the i-th electrical stimulation is p_i. Due to the phenomenon of facilitation or depletion, the p_i will change with the variation of i under continuous stim training conditions.

$$k_i \sim Binomial(N, p_i)$$

$$Var(k_i) = N \cdot p_i \cdot (1 - p_i)$$

η_i is the measurement noise, and we believe that the measurement noise follows an independent normal distribution with a mean of 0. In the actual experimental results, we believe that the operability of η_i is defined as the noise recorded by the experimental instrument in a resting state.

$$\eta_i \sim Normal(0, \sigma_\eta^2)$$

Moment Information

First-Order Moment (Mean)

Since we aim to perform calculations for each individual synapse, all sweeps from a single cell are treated as one dataset for analysis, from which first-order and second-order moment information is extracted.

The first-order moment is the mean. During theoretical derivation, since the random variable η_i follows a normal distribution with a mean of 0, the random term in the mean is approximately eliminated:

$$\mu_i = E(A_i) = E(q_{sweep}) \cdot E(k_i) + E(\eta_i) = q \cdot (N \cdot p_i) + 0$$

For convenience in subsequent substitutions, the above formula can be transformed into:

$$\frac{\mu_i}{q} = N \cdot p_i$$

Second-Order Moment (Covariance)

The second-order moment is the covariance:

$$Cov(A_i, A_j) = Cov(q_{sweep} \cdot k_i + \eta_i, q_{sweep} \cdot k_j + \eta_j)$$

https://doi.org/10.3390/xxxxx

Since η_i and η_j are independent random variables, their contribution to the covariance result is 0, so this term can be directly eliminated later. In covariance calculation, we treat the $sweep$ as the independent variable and A_i as the dependent variable. Therefore, the covariance expands as:

$$Cov(A_i, A_j) = \frac{\sum_{sweep=1}^{T}(q_{sweep} \cdot k_i - q \cdot k_i)(q_{sweep} \cdot k_j - q \cdot k_j)}{T}$$
$$= \frac{\sum_{sweep=1}^{T}(q_{sweep}^2 k_i k_j - 2q_{sweep} q k_i k_j + q^2 k_i k_j)}{T}$$
$$= E(q_{sweep}^2 \cdot k_i \cdot k_j) - E(q_{sweep} \cdot k_i) \cdot E(q_{sweep} \cdot k_j)$$

Since $q_{sweep} \sim Normal(q, \sigma_q^2)$, the expected variance of a normally distributed random variable $E(q_{sweep}^2) = q^2 + \sigma_q^2$, the above equation can be expanded to:

$$Cov(A_i, A_j) = (q^2 + \sigma_q^2) \cdot E(k_i) \cdot E(k_j) - q^2 \cdot E(k_i) \cdot E(k_j)$$
$$= \sigma_q^2 \cdot E(k_i) \cdot E(k_j)$$

And due to the properties of the binomial distribution, $k_i \sim Binomial(N, p_i)$, $E(k_i) = Np_i$, so the above equation can be rewritten as:

$$Cov(A_i, A_j) = \sigma_q^2 \cdot (Np_i) \cdot (Np_j)$$

To minimize the influence of the difficult-to-calculate p, we substitute using the mean current formula $\frac{\mu_i}{q} = N \cdot p_i$:

$$Cov(A_i, A_j) = \sigma_q^2 \cdot \frac{\mu_i}{q} \cdot \frac{\mu_j}{q} = \sigma_q^2 \frac{\mu_i \cdot \mu_j}{q^2}$$

Second-Order Moment (Variance)

When the two random variables in the covariance are the same, it becomes the variance. The variance expansion is based on the law of total variance:

$$i = j, \qquad Cov(A_i, A_j) = Var(A_i) = E[Var(A_i | q_{sweep})] + Var[E(A_i | q_{sweep})]$$

Within each $sweep$, $(A_i | q_{sweep}) = q_{sweep} \cdot k_i + \eta_i$, and within a $sweep$, q_{sweep} is considered a fixed constant, while between sweeps, q_{sweep} is a random variable. Therefore, when we want to find the combined result for all sweeps of a synapse:

$$Var(A_i | q_{sweep}) = q_{sweep}^2 \cdot Var(k_i) + \sigma_\eta^2$$
$$E[Var(A_i | q_{sweep})] = E(q_{sweep}^2) \cdot [N \cdot p_i \cdot (1 - p_i)] + 0 + \sigma_\eta^2$$
$$= (q^2 + \sigma_q^2) \cdot [N \cdot p_i \cdot (1 - p_i)] + \sigma_\eta^2$$
$$= q^2 \cdot N \cdot p_i \cdot (1 - p_i) + \sigma_q^2 \cdot N \cdot p_i \cdot (1 - p_i) + \sigma_\eta^2$$

In the other term of the law of total variance:

$$E(A_i | q_{sweep}) = q_{sweep} \cdot (N \cdot p_i)$$
$$Var[E(A_i | q_{sweep})] = \sigma_q^2 \cdot N^2 \cdot p_i^2$$

Finally, we obtain the expanded formula for the variance:

$$Var(A_i) = q^2 \cdot N \cdot p_i \cdot (1 - p_i) + \sigma_q^2 \cdot N \cdot p_i \cdot (1 - p_i) + \sigma_q^2 \cdot N^2 \cdot p_i^2 + \sigma_\eta^2$$
$$= q^2 \cdot N \cdot p_i \cdot (1 - p_i) + \sigma_q^2 [N \cdot p_i \cdot (1 - p_i) + N^2 \cdot p_i^2] + \sigma_\eta^2$$

Simplify irretrievable relationships

Elimination of $N^2 \cdot p_i^2$

The current equations contain numerous variables that cannot be directly observed. To reduce their number, we need to perform relevant substitutions or transformations using known formulas.

To eliminate the computationally challenging term $N^2 \cdot p_i^2$, we multiply $Cov(A_i, A_j)$ by an observable coefficient and subtract it from $Var(A_i)$. thereby canceling this term. Consequently, we define a fully observable equation $F_{i,j}$:

$$F_{i,j} = Var(A_i) - \frac{\mu_i}{\mu_j} Cov(A_i, A_j) - \sigma_\eta^2$$

Expand and simplify the above formula

$$F_{i,j} = q^2 \cdot N \cdot p_i \cdot (1 - p_i) + \sigma_q^2 \cdot N \cdot p_i \cdot (1 - p_i) + \sigma_q^2 \cdot N^2 \cdot p_i^2 + \sigma_\eta^2 - \frac{\mu_i}{\mu_j} \cdot \sigma_q^2 \cdot (Np_i) \cdot (Np_j) - \sigma_\eta^2$$

Coincidentally, $\frac{\mu_i}{\mu_j} = \frac{p_i}{p_j}$, so we can substitute it to obtain:

$$F_{i,j} = q^2 \cdot N \cdot p_i \cdot (1 - p_i) + \sigma_q^2 \cdot N \cdot p_i \cdot (1 - p_i) + \sigma_q^2 \cdot N^2 \cdot p_i^2 + \sigma_\eta^2 - \frac{p_i}{p_j} \cdot \sigma_q^2 \cdot (Np_i) \cdot (Np_j) - \sigma_\eta^2$$

$$= (q^2 + \sigma_q^2) \cdot N \cdot p_i \cdot (1 - p_i)$$

$$F_{i,j} = E(q_{sweep}^2) \cdot N \cdot p_i \cdot (1 - p_i)$$

Eliminate $\boldsymbol{p_i}$

Our primary objective is to estimate q as the first step; therefore, we aim to eliminate the stimulus-dependent term p_i whenever possible. Using the substitution $p_i = \frac{\mu_i}{qN}$ we rewrite

$$F_{i,j} = E(q_{sweep}^2)(\frac{\mu_i}{q} - \frac{\mu_i^2}{q^2 N})$$

Through straightforward algebraic manipulation, we isolate the term containing N:

$$\frac{\mu_i^2}{q^2 N} = \frac{\mu_i}{q} - \frac{F_{i,j}}{E(q_{sweep}^2)}$$

In principle, any pair of distinct ordered indices i, j, k, l can be used to eliminate N by multiplying with known coefficients involving μ. For instance, consider the following pair of equations:

$$\begin{cases} \frac{\mu_i^2}{q^2 N} = \frac{\mu_i}{q} - \frac{F_{i,j}}{E(q_{sweep}^2)} \\ \frac{\mu_k^2}{q^2 N} = \frac{\mu_k}{q} - \frac{F_{k,l}}{E(q_{sweep}^2)} \end{cases}$$

Multiplying the first equation by μ_k^2 and the second by μ_i^2, then subtracting the two, yields

$$\mu_k^2(\frac{\mu_i}{q} - \frac{F_{i,j}}{E(q_{sweep}^2)}) = \mu_i^2(\frac{\mu_k}{q} - \frac{F_{k,l}}{E(q_{sweep}^2)})$$

Multiplying both sides by $E(q_{sweep}^2)$ and rearranging for q, we obtain

$$\mu_k^2(\mu_i E(q_{sweep}^2) - qF_{i,j}) = \mu_i^2(\mu_k E(q_{sweep}^2) - qF_{k,l})$$

To solve q, we will simply transform this equation,

$$q = \frac{\mu_k \mu_i E(q_{sweep}^2)(\mu_k - \mu_i)}{\mu_k^2 F_{i,j} - \mu_i^2 F_{k,l}}$$

Eliminate $\boldsymbol{\sigma_q^2}$

Branch 1: Rapid Approximation

For a simplified estimation, if the data suggest that q is highly stable such that $\sigma_q^2 \to 0$, then

$$E(q_{sweep}^2) \approx q^2$$

Under this condition,

$$q \approx \frac{\mu_k^2 F_{i,j} - \mu_i^2 F_{k,l}}{\mu_k \mu_i (\mu_k - \mu_i)}$$

Based on this estimate, both N and p_i can subsequently be computed:

$$N \approx \frac{\mu_i^2}{\mu_i q - F_{i,j}}$$

$$\widehat{p_i} = \frac{\mu_i}{qN}$$

Branch 2: Detailed Computation

If σ_q^2 cannot be neglected or is known to exhibit substantial variability, a more explicit treatment using covariance is required:

$$Cov(A_i, A_j) = \frac{\sigma_q^2}{q^2} \mu_i \cdot \mu_j$$

For convenience of notation, define

$$R_{i,j} = \frac{\sigma_q^2}{q^2} = \frac{Cov(A_i, A_j)}{\mu_i \cdot \mu_j}$$

Since many index pairs i, j exist in the dataset, we estimate a more robust $\hat{R}$ using the empirical mean:

$$\hat{R} = E(\frac{Cov(A_i, A_j)}{\mu_i \cdot \mu_j}) = \frac{1}{C_T^2} \cdot \sum_{i \neq j}^{C_T^2} \frac{Cov(A_i, A_j)}{\mu_i \cdot \mu_j}$$

Using $\hat{R}$, we can compute the previously unobtainable second moment:

$$E(q_{sweep}^2) = \hat{q}^2 \cdot (1 + \hat{R})$$

Substituting this $E(q_{sweep}^2)$ into the expression for q, we obtain

$$\hat{q} = \frac{\mu_k \mu_i \hat{q}^2 \cdot (1 + \hat{R})(\mu_k - \mu_i)}{\mu_k^2 F_{i,j} - \mu_i^2 F_{k,l}}$$

which simplifies to:

$$\hat{q} = \frac{\mu_k^2 F_{i,j} - \mu_i^2 F_{k,l}}{\mu_k \mu_i (1 + \hat{R})(\mu_k - \mu_i)}$$

Similarly, we derive estimates for $\hat{N}$ and $\hat{p_i}$. Starting from the rearranged form of $F_{i,j}$:

$$F_{i,j} = E(q_{sweep}^2)(\frac{\mu_i}{\hat{q}} - \frac{\mu_i^2}{\hat{q}^2 \hat{N}})$$

we obtain

$$\frac{F_{i,j}}{E(q_{sweep}^2)} = \frac{\mu_i}{\hat{q}} - \frac{\mu_i^2}{\hat{q}^2 \hat{N}}$$

$$\frac{\mu_i^2}{\hat{q}^2 \hat{N}} = \frac{\mu_i}{\hat{q}} - \frac{F_{i,j}}{E(q_{sweep}^2)}$$

$$\frac{1}{\hat{N}} = \frac{\hat{q}^2}{\mu_i^2} \cdot (\frac{\mu_i}{\hat{q}} - \frac{F_{i,j}}{E(q_{sweep}^2)})$$

$$= \frac{\hat{q}}{\mu_i} - \frac{\hat{q}^2}{\mu_i^2} \cdot \frac{F_{i,j}}{E(q_{sweep}^2)}$$

and therefore

$$\hat{N} = \frac{1}{\frac{\hat{q}}{\mu_i} - \frac{\hat{q}^2}{\mu_i^2} \cdot \frac{F_{i,j}}{E(q_{sweep}^2)}}$$

Substituting $E(q_{sweep}^2) = q^2 \cdot (1 + \hat{R})$:

$$\hat{N} = \frac{1}{\frac{\hat{q}}{\mu_i} - \frac{\hat{q}^2}{\mu_i^2} \cdot \frac{F_{i,j}}{q^2 \cdot (1 + \hat{R})}}$$

$$= \frac{1}{\frac{\hat{q}}{\mu_i} - \frac{F_{i,j}}{\mu_i^2 \cdot (1 + \hat{R})}}$$

$$= \frac{\mu_i^2}{\mu_i \hat{q} - \frac{F_{i,j}}{(1 + \hat{R})}}$$

Finally, the release probability $\hat{p_i}$ estimate follows directly:

$$\hat{p_i} = \frac{\mu_i}{\hat{q} \hat{N}}$$

Parameter Optimization

At this stage, all synaptic parameters of interest can be computed using the preceding derivations. Specifically,

$$\begin{cases} \hat{q} = \dfrac{\mu_k^2 F_{i,j} - \mu_i^2 F_{k,l}}{\mu_k \mu_i (1+\hat{R})(\mu_k - \mu_i)} \\ \hat{N} = \dfrac{\mu_i^2}{\mu_i \hat{q} - \dfrac{F_{i,j}}{(1+\hat{R})}} \\ \hat{p_i} = \dfrac{\mu_i}{\hat{q}\hat{N}} \end{cases}$$

If higher accuracy is desired, one may compute multiple estimates using different index tuples (i,j,k,l) and subsequently average the results, thereby reducing estimation error as much as possible. In calculating $\hat{q}$, the index constraints $i \neq j$, $i \neq k$ and $k \neq l$ must be satisfied.

$$\begin{cases} E(\hat{q}) = \dfrac{1}{C_T^2 \cdot C_{T-1}^2} \displaystyle\sum_{i \neq j}^{C_T^2} \sum_{k \neq i \,\&\, k \neq l}^{C_{T-1}^2} \hat{q}_{i,j,k,l} \\ \hat{N} = \max_{\hat{N}_{i,j} \in \mu \pm 3\sigma} \hat{N}_{i,j} \\ \hat{p_i} = \dfrac{\mu_i}{\hat{q}\hat{N}} \end{cases}$$

Because biological data typically exhibit substantial stochastic variability, occasional negative q values may arise. Such results are considered unreliable outliers caused by excessive experimental noise and should be excluded when computing the mean estimate.

Regarding the estimation of N, covariance-based estimators tend to be biased downward, and biologically only a small portion of the over-saturated vesicle pool contributes to release. Therefore, it is advisable to discard values outside the 3σ range and select the maximum among the remaining estimates.

This yields more stable and accurate estimates of $E(\hat{q})$, $\hat{N}$ and $\hat{p_i}$.

Section S3 Error Analysis under Non-Ideal Neuronal Conditions

In real synapses, release sites are heterogeneous in both quantal size (q) and release probability (p). This heterogeneity arises from several biological factors, including variability in vesicle neurotransmitter content, differences in postsynaptic receptor density, spatial differences between release sites and receptors, and variability in presynaptic Ca^{2+} channel coupling. Consequently, different release sites within a single synapse may exhibit distinct quantal amplitudes and release probabilities, which deviates from the idealized assumption of identical q and p in classical quantal models.

In this scenario, how does the error of the parameter estimates behave when using a moment-based (first- and second-order) approach? The impact of such errors can be analyzed through straightforward analytical derivations.

We first consider the case where heterogeneity arises in the quantal size q. Specifically, we assume that the quantal size across release sites follows a normal distribution with mean q. Under this assumption, it can be readily inferred that the overall variance of the observed data will increase, while the covariance structure remains unchanged at the leading order.

To facilitate the derivation, consider two sets of EPSC measurements (for example, corresponding to stimulus indices 1 and 4). For simplicity, we denote these two random variables as X and Y, respectively.

$$X = (x_1, x_2, \ldots, x_n), \qquad Y = (y_1, y_2, \ldots, y_n)$$

Now add an independent random perturbation to each data:

$$X_i' = x_i + \varepsilon_i, \qquad Y_i' = y_i + \delta_i$$

where

$$\varepsilon_i \sim N(0, \sigma_\varepsilon), \qquad \delta_i \sim N(0, \sigma_\delta),$$

ε_i, δ_i are mutually independent and also independent of the original data X, Y。

Regarding the changes in variance,

$$Var(X') = Var(X) + Var(\varepsilon) = Var(X) + \sigma_\varepsilon^2$$
$$Var(Y') = Var(Y) + \sigma_\delta^2$$

As for the change in covariance,

$$Cov(X', Y') = Cov(X + \varepsilon, Y + \delta)$$

Under the current assumptions, covariance satisfies bilinearity, so it can be directly decomposed as:

$$Cov(X', Y') = Cov(X, Y) + Cov(X, \delta) + Cov(\varepsilon, Y) + Cov(\varepsilon, \delta)$$

However, the random perturbations ε and δ are independent random variables and thus orthogonal to all other variables, implying

$$\begin{cases} Cov(X, \delta) = 0 \\ Cov(\varepsilon, Y) = 0 \\ Cov(\varepsilon, \delta) = 0 \end{cases}$$

Therefore,

$$Cov(X', Y') = Cov(X, Y)$$

In summary, it follows straightforwardly that, in EPSC results, when heterogeneity in q occurs across loci, the theoretical variance increases while the covariance remains unchanged.

However, the above scenario remains overly idealized. We now consider a more general and realistic case in which both q and p exhibit heterogeneity across release sites, leading to variability in the EPSC response following each stimulus. Before proceeding with the formal derivation, it is instructive to briefly consider the potential biological origins of such heterogeneity, which will guide the formulation of the model.

Within a single neuron, differences in synaptic activity levels may result in local variations in vesicle recycling efficiency, thereby affecting the "maturity" or size of vesicles across release sites. Nevertheless, although the number of neurotransmitter molecules may fluctuate across sites, the expectation remains unchanged. We therefore assume that the quantal size follows a normal distribution across release sites with mean $\bar{q}$, i.e.,

$$q_r \sim N\left(\bar{q}, \sigma_{q,site}^2\right)$$

In addition, the release probability is strongly influenced by the local calcium concentration near each release site. Variations in the distance between release sites and calcium channels can introduce site-dependent offsets in release probability. Accordingly, we define the release probability at site r under the i-th stimulus as

$$p_{r,i} = p_r + \delta_r, \qquad \delta_r \sim N\left(0, \sigma_p^2\right)$$

We further assume that q_r and δ_r are independent, and that δ_r represents a fixed offset for each site across different stimuli.

Under these assumptions, the total response to the i-th stimulus can be written as

$$A_i = \sum_{r=1}^{N} q_r X_{r,i} + \eta_i, \qquad X_{r,i} \sim Binomial(p_i + \delta_r)$$

We first analyze the behavior of the mean. When $E(\delta_r) = 0$, and q_r and δ_r are independent, we obtain

$$\mu_i = E(A_i) = \sum_{r=1}^{N} E(q_r)E(X_{r,i}) = \sum_{r=1}^{N} E(q_r)E(p_i + \delta_r) = N\bar{q}p_i$$

Thus, the mean remains identical to that predicted by the idealized model. This indicates that the effects of random heterogeneity do not influence the first-order moment, but instead manifest in higher-order statistics.

Based on the above reasoning, since the random components are mutually independent, and assuming a sufficiently large sample size, we can approximate that the covariance $Cov(A_i, A_j)$ also remains unchanged. Consequently, the key quantity used in the original derivation,

$$R_{i,j} = \frac{Cov(A_i, A_j)}{\mu_i \cdot \mu_j}$$

is likewise preserved.

However, the variance increases under this heterogeneous setting.

$$Var(A_i) = \sum_{r=1}^{N} q_r^2 p_{r,i}(1 - p_{r,i}) + \sigma_\eta^2$$

$$= \sum_{r=1}^{N} \bar{q}^2 p_{r,i}(1 - p_{r,i}) + \sum_{r=1}^{N} \sigma_{q,site}^2 p_{r,i}(1 - p_{r,i}) + \sigma_\eta^2$$

The key intermediate observable $F_{i,j}$, after averaging across release sites, will increase as a result of the increase in $Var(A_i)$, since the mean and covariance terms in its definition remain approximately unchanged:

$$F_{i,j} = Var(A_i) \uparrow - \frac{\mu_i}{\mu_j} Cov(A_i, A_j) - \sigma_\eta^2$$

However, an interesting complication arises at this point. Since the final estimator $\hat{q}$ is computed as the difference between two terms involving $F_{i,j}$, it becomes difficult to directly determine the direction of change in $\hat{q}$:

$$\hat{q} = \frac{\mu_k^2 F_{i,j} \uparrow - \mu_i^2 F_{k,l} \uparrow}{\mu_k \mu_i (1 + \hat{R})(\mu_k - \mu_i)}$$

Even if we substitute the analytical expression of $F_{i,j}$ into the above equation and fully expand it, the resulting expression remains too complex to directly infer the trend of $\hat{q}$:

$$\hat{q} = \frac{\bar{q}^2 + \sigma_{q,site}^2}{\bar{q}(1 + \hat{R})} \left[1 - \sigma_p^2 \left(\frac{1}{p_i} + \frac{1}{p_k}\right)\right] - \frac{\frac{p_k}{p_j} Cov(A_i, A_j) - \frac{p_i}{p_l} Cov(A_k, A_l)}{N\bar{q}(1 + \hat{R})(p_k - p_i)}$$

Nevertheless, a simple approximation can be made. Since the estimator $\hat{q}$ is essentially constructed as a ratio, we can express it in the following approximate form:

$$\hat{q} \approx \frac{f(q^{n+1})}{g(q^n)} + b_{i,j,k,l}$$

where $f(x)$ and $g(x)$ are linear functions that preserve the highest-order term of the expression, and $b_{i,j,k,l}$ is a random constant determined by the selected index combination.

From the fully expanded expression, it can be observed that the highest-order term in the numerator of $\hat{q}$ is quadratic. This implies that $f(x)$ is effectively a second-order function, while $g(x)$ is first-order.

Therefore, the dominant behavior of $\hat{q}$ can be approximated as

$$\hat{q} \propto \frac{\bar{q}^2 + \sigma_{q,site}^2}{\bar{q}(1 + \hat{R})}$$

As long as $\sigma_{q,site}^2 > 0$, the presence of heterogeneity in q will lead to an upward bias in $\hat{q}$. This bias scales approximately linearly with $\bar{q}$, but is constrained by the denominator $\bar{q}(1 + \hat{R})$, resulting in a relatively small overall deviation.

Similarly, when σ_p^2 is nonzero, the multiplicative term

$$1 - \sigma_p^2 \left(\frac{1}{p_i} + \frac{1}{p_k}\right)$$

introduces a downward bias in the estimation of q. This effect is also approximately linear in magnitude.

In addition, the effects on $\hat{N}$ and $\hat{p_i}$ are considerably more difficult to derive analytically. We therefore refer to simulation results for a more detailed characterization of their behavior.

Based on the above analysis, we can obtain the following qualitative conclusions:

1.When heterogeneity exists in q across release sites, the estimated $\hat{q}$ tends to be slightly biased upward.

2. When heterogeneity exists in p across release sites, the estimated $\hat{q}$ exhibits a very slight downward bias. Under the parameter regimes typically used in this study (e.g., $\bar{q} = 10$, $\sigma_{q,site} = 4$, $\sigma_p = 0.05$), this effect is approximately one to two orders of magnitude smaller than the bias induced by heterogeneity in q.

The corresponding simulation results are presented in Figure 7 of the main text, showing good qualitative agreement with the analytical estimates.

Section S4 Error analysis under non Gaussian noise conditions

In practice, noise is often implicitly assumed to be Gaussian due to its prevalence and analytical convenience. However, there is no guarantee that experimentally observed noise strictly follows a Gaussian distribution, especially when systematic noise is introduced by measurement devices or environmental factors. Therefore, in this section, we analytically investigate how non-Gaussian noise may affect the overall parameter estimation results.

Noise Assumption

We assume that the noise follows an arbitrary (non-Gaussian) distribution, while maintaining finite expectation and variance:

$$\eta_i \sim arbitrary\ distribution, \qquad E(\eta_i) = m_\eta, \qquad Var(\eta_i) = \sigma_\eta^2$$

We further allow for cross-stimulus correlations:

$$Cov(\eta_i, \eta_j) = \gamma_{ij}, \qquad (i \neq j)$$

In short, this is a case that is "non-Gaussian, yet has a computable expectation, finite variance, and well-defined temporal correlation."

First Moment

We first compute the expectation of the EPSC:

$$E(A_i) = E(q_{sweep}k_i + \eta_i)$$

Expanding under the previous assumptions yields:

$$\mu_i = qNp_i + m_\eta$$

If the noise has zero mean, i.e., $m_\eta = 0$, the expression remains unchanged. Otherwise, a nonzero expectation introduces a direct shift in μ_i.

Second Moment

We next examine the covariance. Expanding the covariance expression gives:

$$Cov(A_i, A_j) = Cov(q_{sweep}k_i, q_{sweep}k_j) + Cov(q_{sweep}k_i, \eta_j) + Cov(\eta_i, q_{sweep}k_j) + Cov(\eta_i, \eta_j)$$

Since the noise is independent of the intrinsic synaptic release process, the cross terms vanish, leading to:

$$Cov(A_i, A_j) = Cov(q_{sweep}k_i, q_{sweep}k_j) + \gamma_{ij}$$

Under sufficiently large sample size, the dependence between k_i and k_j becomes negligible, and we obtain:

$$Cov(q_{sweep}k_i, q_{sweep}k_j) \approx \sigma_q^2 N^2 p_i p_j$$

$$Cov\left(A_i, A_j\right) \approx \sigma_q^2 N^2 p_i p_j + \gamma_{ij}$$

$$\approx \frac{\sigma_q^2}{q^2}\mu_i\mu_j + \gamma_{ij}$$

If $m_\eta = 0$ and the noise is independent (i.e., $\gamma_{ij} = 0$), the covariance expression remains unchanged. However, when $\gamma_{ij} \neq 0$, it will directly affect the estimation of R_{ij}.

$$R_{ij} = \frac{Cov\left(A_i, A_j\right)}{\mu_i\mu_j}$$

Substituting the above result yields:

$$R_{ij} = \frac{\sigma_q^2}{q^2} + \frac{\gamma_{ij}}{\mu_i\mu_j}$$

Thus, the direction and magnitude of deviation in R_{ij} depend on the sign and scale of γ_{ij}.

Next, we examine the variance. Substituting the new noise model into the original variance expression yields:

$$Var(A_i) = (q^2 + \sigma_q^2)Np_i(1 - p_i) + \sigma_q^2 N^2 p_i^2 + \sigma_\eta^2$$

Notably, this expression is identical to the original variance formula. Therefore, the distributional form of the noise does not affect the variance computation.

Key Intermediate Variables $\boldsymbol{F_{ij}}$

Similarly, substituting the non-Gaussian noise into the original derivation of F_{ij} gives:

$$F_{ij} = (q^2 + \sigma_q^2)Np_i(1 - p_i) - \frac{p_i}{p_j}\gamma_{ij}$$

If $\gamma_{ij} = 0$, the quantity F_{ij} remains unchanged. Otherwise,

$$F_{ij}^{obs} = F_{ij}^{ideal} - \frac{p_i}{p_j}\gamma_{ij}$$

The Impact On Parameter Estimation

Using the full analytical expression, the observed estimator becomes:

$$\hat{q}_{obs} = \frac{\mu_k^2(F_{i,j}^{ideal} - \frac{p_i}{p_j}\gamma_{ij}) - \mu_i^2(F_{k,l}^{ideal} - \frac{p_k}{p_l}\gamma_{kl})}{\mu_k\mu_i(1 + \hat{R}_{obs})(\mu_k - \mu_i)}$$

To quantify the deviation, we define:

$$\Delta q = \hat{q}_{obs} - \hat{q}$$

An approximate expression can be written as:

$$\Delta q \approx -\frac{\mu_k^2\frac{p_i}{p_j}\gamma_{ij} - \mu_i^2\frac{p_k}{p_l}\gamma_{kl}}{\mu_k\mu_i(1 + \hat{R}_{obs})(\mu_k - \mu_i)} - \hat{q}\frac{\frac{\gamma_{ij}}{\mu_i\mu_j}}{1 + R_{ij}}$$

If the noise is non-Gaussian but uncorrelated, i.e.,

$$\gamma_{ij} = 0, \qquad F_{ij}^{obs} = F_{ij}^{ideal}, \qquad R_{ij}^{obs} = R_{ij}^{ideal}$$

and therefore:

$$\Delta q = 0$$

In this case, $\hat{q}, \hat{N}, \hat{p_i}$ are not affected by systematic bias.

However, if the non-Gaussian noise exhibits cross-stimulus correlation ($\gamma_{ij} \neq 0$), all estimated parameters $\hat{q}, \hat{N}$, and $\hat{p_i}$ will exhibit systematic deviations.

Conclusion

It can be readily seen that whether random noise affects parameter estimation is not fundamentally determined by whether the noise is Gaussian. Instead, the critical factors are whether the noise has a nonzero mean and whether it introduces cross-stimulus correlations.

https://doi.org/10.3390/xxxxx
